# Life-History Parameters of *Phyllotreta striolata* (F.) (Coleoptera: Chrysomelidae) Acquired by a Laboratory-Rearing Method

**DOI:** 10.3390/insects16030260

**Published:** 2025-03-03

**Authors:** Yiyun Wei, Jincheng Zhou, Bin Chen, Panpan Tian, Chen Luo

**Affiliations:** 1 Chongqing Key Laboratory of Vector Control and Utilization, Chongqing Normal University, Chongqing 401331, China; weiyiyun1988@163.com (Y.W.); bin.chen@cqnu.edu.cn (B.C.); 2 Institute of Plant Protection, Beijing Academy of Agriculture and Forestry Sciences, Beijing 100097, China; 3 College of Plant Protection, Shenyang Agricultural University, Shenyang 110866, China; parasitoidswasp@163.com; 4 College of Plant Protection, Yangzhou University, Yangzhou 225009, China

**Keywords:** *Phyllotreta striolata*, laboratory-rearing method, life-history parameters

## Abstract

*Phyllotreta striolata* (Fabricius) (Coleoptera: Chrysomelidae) is one of the most damaging pests affecting vegetables. This pest feeds on the leaves and roots of Brassicaceae plants during their adult and larval stages, respectively, resulting in substantial economic losses. The laboratory-rearing methods for *P. striolata* and its life-history parameters have not been fully studied. This study developed a laboratory method for the generational rearing of *P. striolata*, including techniques for egg collection and rearing immature individuals, which allows for the continuous observation and collection of individuals at various developmental stages. This method established five F1 populations and three consecutive generations of *P. striolata* from various field-collected parent populations. During the rearing process, developmental durations and survival rates at four stages of *P. striolata* were acquired, confirming the method’s effectiveness. The female ratio, tibia length of the hind leg, and fluctuating asymmetry of adults in three of the five F1 populations were measured to assess *P. striolata*’s adaptability to the rearing conditions. The established laboratory-rearing method and life-history parameters of *P. striolata* could potentially contribute to research in its ecology, population dynamics prediction, and pest management.

## Simple Summary

*Phyllotreta striolata* (Fabricius) (Coleoptera: Chrysomelidae) is a significant pest for vegetable crops, with its larvae and adults feeding on the roots and leaves of Brassicaceae plants, respectively, resulting in severe yield losses. The laboratory-rearing and life-history parameters of *P. striolata* are still poorly understood. This study established a laboratory-rearing method for *P. striolata*, which introduces an egg collection technique and a rearing approach for immature insects that facilitated the real-time observation and collection of individuals across various developmental stages. This method obtained the first filial (F1) populations from field parents and their subsequent generations. The developmental durations and survival rates of *P. striolata* at different life stages were documented. In three of the five F1 populations, the sex ratio and fluctuating asymmetry based on the tibial length of adults were measured, indicating the similar adaptability of *P. striolata* to the rearing conditions. This study can lay a foundation for research on the ecology, population dynamics, and pest management strategies of *P. striolata*.

## 1. Introduction

The striped flea beetle, *Phyllotreta striolata* (Fabricius) (Coleoptera: Chrysomelidae), is a significant pest of Brassicaceae vegetables worldwide, particularly in Asia [1,2], North America [3,4], and Europe [5]. In recent decades, *P. striolata* has been reported in vegetable fields in southern China, leading to yield losses and economic damage [1,6,7,8]. The lifecycle of *P. striolata* includes immature stages (egg, larva, pupa) residing in soil and adults living on the ground [3,5,7,8]. The adults and larvae of *P. striolata* feed on the leaves and roots of Brassicaceae plants, respectively [3,9,10,11,12]. Brassicaceae hosts such as *Brassica pekinensis*, *B. juncea*, *B. rapa*, *B. napus*, and *Raphanus sativus* are frequently devoured, resulting in leaf holes, root damage, and seedling devastation [1,3,12,13,14]. The larvae can damage the root epidermis, triggering root rot, resulting in plant death [12,13]. Furthermore, a significant intergenerational overlap of *P. striolata* exacerbates pest control challenges in the field. Dynamic monitoring of the *P. striolata* population and pest management are important research areas [2,6,12,14,15,16]. Monitoring and damage assessment of *P. striolata* is primarily conducted by trap placement, quantification of defoliation, and comprehensive analysis of big data [4,8,15,16]. Management strategies for *P. striolata* include agricultural regulation [12,17], biological control [18,19,20], chemical control [14,21,22], and integrated tactics [12,15,23].

Studying the life-history parameters of insects is crucial for multiple scientific fields [24,25]. Understanding these parameters facilitates in-depth research into the ecology, physiology, behavior, pesticide resistance, and insect–host relationships [24]. Life-history parameters include the development durations, survival rates, adult fecundity, and other functional traits, which are quantified and analyzed within insect life tables [24,25,26,27]. The sex ratio of populations is an important parameter for studying population dynamics and adaptability [24,27], while fluctuating asymmetry (FA) can reflect the impact of environmental pressures on population adaptability [28]. Utilizing age–stage life tables to understand the population structure of pests, predict population dynamics, and detect changes in their susceptibility to pesticides is imperative for developing effective control strategies [24]. As ectothermic organisms, the life history traits of insects are influenced by various biotic and abiotic factors [25], with environmental characteristics based on geographical origin being an important factor affecting life-history parameters [29,30]. Studies have shown that under the same laboratory-rearing conditions, different geographical populations often exhibit different life-history parameters. For instance, populations of *Sitobion avenae* (F.) (Hemiptera: Aphididae) from both sides of the Qinling Mountains in China differ in nymphal development period, adult fecundity, and lifespan [31]. Similarly, five different geographical populations of *Plutella xylostella* (L.) (Lepidoptera: Plutellidae) in China show variations in female fecundity, adult longevity, and mean generation time [29]. Six geographical populations of *Rhopalosiphum padi* (L.) (Hemiptera: Aphididae) in China exhibit differences in nymphal mortality, development time, and mean fecundity [30], and different geographical populations of *Musca domestica* (L.) (Diptera: Muscidae) in Pakistan vary in immature duration, egg hatching, eclosion rate, and fecundity [32]. Researchers believe that these differences are primarily due to genetic adaptations among populations and local environmental characteristics. By collecting and rearing different geographical populations of *P. striolata* indoors, it is possible to obtain the life-history parameters of different populations, providing a foundation for revealing geographical factors contributing to population differences, which is crucial for developing control strategies for populations in different regions [29,30]. Also, establishing a reliable laboratory method for rearing a population of *P. striolata* is of paramount importance.

Rearing methods for *P. striolata* have been developed over the past few decades [33,34,35,36,37,38,39]. In the studies conducted by Beran et al. [38], for analyzing the accumulation of host plant glucosinolates in adults, field-collected *P. striolata* adults were reared on *Sinapis alba*, *Brassica rapa*, *Arabidopsis thaliana*, and *B. juncea*. Tansey et al. reared adults on potted canola seedlings treated with insecticides within mesh cages to evaluate the response of *P. striolata* [14]. For a bioassay targeting a pivotal gene of *P. striolata* using dsRNA, Zhao et al. reared adults in culture bottles containing Chinese cabbage seedlings with fine soil [36]. Due to research purposes, these studies focus solely on the rearing of adult *P. striolata*, without the requirement for immature individuals. In one study by Xian et al. [35], adults were placed into an oviposition bottle lined with black cloth, containing choy sum seedlings, for egg collection. *Phyllotreta striolata* adults were also reared in oviposition tubs containing cabbage leaves, and eggs were collected from the paper towels attached to the tubs in one other study [36]. In both experiments, researchers relocated the eggs next to germinating seedlings in sandy soil or into the soil fissures at the base of the plant stem for subsequent developmental stages [35,36]. Neither of the studies requires the observation and collection of individuals at a certain age; however, pathogens present in the soil could infect immature *P. striolata* if no sterilization is performed. Fresh *Brassica rapa* leaves were also used as oviposition substrates and larval food in a Petri dish in the study by Patricio and Ocampo [37]. This method is not usual due to the oviposition habits and larval food preferences of *P. striolata* [3,9,12]. In several previous reports, newly hatched larvae were placed on host plant roots or transferred to artificial grooves within radish roots [33,34]. Regarding potential larvae loss during rearing or mechanical injury, this can happen when transferring them to the new feed in these studies [33,34,36,37]. The Petri dishes containing moist cloth, radish slices, *B*. *rapa* leaves, or plant roots with larvae were used as pupation or eclosion sites [33,34,35,37], where humidity required careful monitoring. Several studies have documented the life-history parameters of *P. striolatas* [33,34,35,36,37]. According to the rearing method of Xian et al. [35], the duration of the larva (including prepupa) and pupa was, respectively, 8.5–12.0 days and 4.0–6.0 days. Nagalingam and Costamagna found the duration of the immature stage of *P. striolata* ranged from 25 to 33 days [36]. Using various rearing methods for *P. striolata*, the pupation rate and eclosion rate were recorded as 83.7–92.7% [33,34,35], and 80.0–97.9% [33,34,35], respectively. However, the life-history parameters of *P. striolata* across different stages still require further study.

Studying laboratory-rearing methods and conditions contributes to understanding the environmental requirements necessary for the growth and development of *P. striolata*, as well as its life-history parameters and behavioral patterns [24,25,36]. On one hand, this knowledge aids in providing test insects for the testing of pest control methods and the exploration of resistance management strategies [24,25]. On the other hand, it provides a foundation for predicting population dynamics and advancing ecological and behavioral-based pest management strategies [24,25,26,27]. In this study, we have developed a laboratory-rearing method for *P. striolata*, inspired by its natural biological characteristics. We have assembled and applied two specialized devices for rearing adults and immature individuals, taking into account their different living habits. Adults feed and oviposit in rearing bottles containing whole host plants, while the immature stages of *P. striolata* are reared in 24-well culture plates. The eggs can be laid on the substrates covering the host plant’s root and develop in the culture wells until adulthood. This method facilitates observing and collecting the larvae and pupae at different developmental stages. Meanwhile, the potential issues arising from insect transfer, feed replacement, and pathogen infection are minimized. Using this method, five F1 populations and three consecutive generations of *P. striolata* from field-collected parents have been obtained while recording durations and survival rates across four developmental stages. We have measured the female ratios, tibia length of the hind leg, and fluctuating asymmetry based on the adults from three of the five F1 populations. The established laboratory-rearing method for *P. striolata*, along with the life-history parameters, can serve as a reference for maintaining indoor strains and provide foundational data for ecological research, population dynamics prediction, and pest management.

## 2. Materials and Methods

### 2.1. Populations

*P. striolata* were collected from Shanghai, Langfang, and Nanjing, China. LF, SH, NJP, NJ10, and NJ11 represent the first filial (F1) populations from five field parental populations in these three cities, successfully established using a laboratory-rearing method developed in this study (Table 1). The five F1 populations, originating from their respective parental populations in Langfang, Shanghai, and Nanjing, were named accordingly. The NJ population and the parental populations of NJ10, NJ11, and NJP are all derived from Nanjing (Table 1). The NJF1, NJF2, and NJF3 populations, representing the first three consecutive filial generations of the NJ population, were established using the laboratory-rearing method developed in this study.

### 2.2. The Laboratory-Rearing Method for P. striolata

#### 2.2.1. The Materials and Equipment

*Brassica rapa var. chinensis* and *Raphanus sativus var. longipinnatus* were selected as the host plants for *P. striolata* adults and larvae due to their food preference [11,38,40]. The *B. rapa* and *R. sativus* were cultivated in a greenhouse. *B. rapa* was used when it reached 8–9 true leaves, and *R. sativus* was used when the diameter of the root exceeded 4 cm. The seeds of *B. rapa* (XIALV No. 2 F_1_) were purchased from Jingyan Yinong (Beijing) Seed Sci-Tech Co., Ltd. (Beijing, China). The seeds of *R. sativus* (SHAWO RADISH SEED) were sourced from Hebei Manchou Agricultural Science and Technology Co., Ltd. (Dingzhou, China).

**Figure 1:**
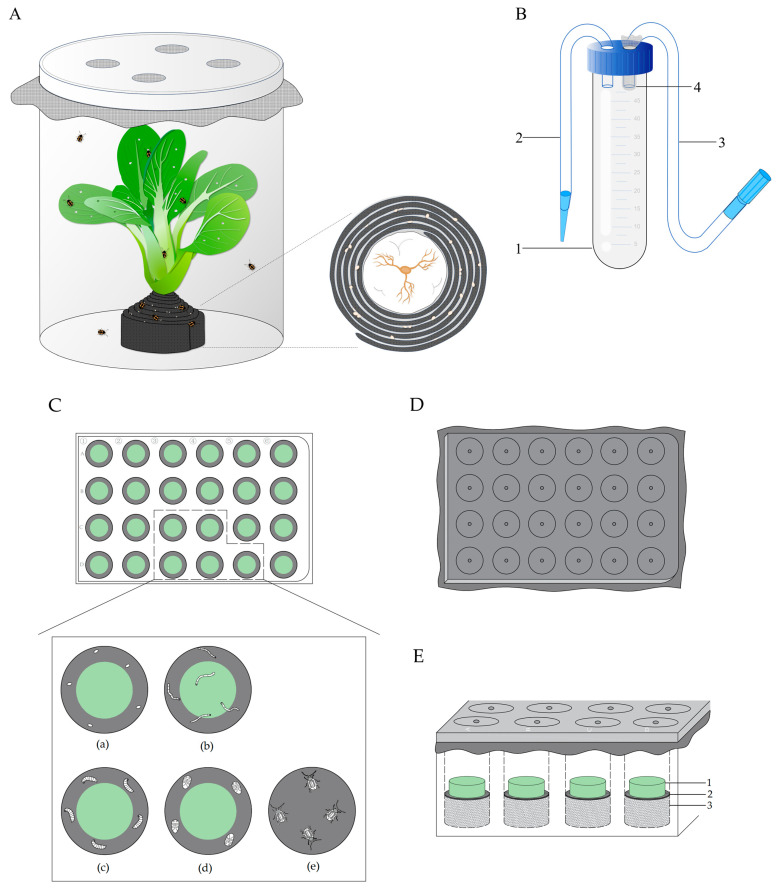
A schematic diagram of the rearing device for *Phyllotreta striolata*. (**A**) The device for adult rearing and oviposition. (**B**) The suction trap device for adult collection: **1**. insect storage tube; **2**. entrance conduit; **3**. negative pressure conduit; **4**. 400-mesh gauze. (**C**) Top view of the rearing device without lid; (**a**) egg, (**b**) larva, (**c**) prepupa, (**d**) pupa, (**e**) adult. (**D**) Top view of the rearing device lid. (**E**) Side view of the rearing device with lid: **1**. round slice of radish root; **2**. round black cotton cloth; **3**. agar substrate.

A transparent feeding bottle was used for adult rearing, with four ventilation holes in the lid, covered with nylon gauze (400 mesh) (Figure 1A). Absorbent cotton was essential for providing water to the host plant within the feeding bottle. A 24-well culture plate (well diameter: 16 mm, depth: 20 mm) from Haimen Haoyuan Experimental Instrument Factory^®^, Haimen, China, was utilized for rearing immature *P. striolata*. To establish a moisturizing and antiseptic substrate within the culture wells, the following materials were utilized: agar powder (Sangon Biotech (Shanghai) Co., Ltd., Shanghai, China), benzylpenicillin sodium (800,000 units, CSPC^®^, Shijiazhuang, China), potassium sorbate (food grade, Solarbio^®^, Beijing, China), and pimaricin (Solarbio^®^). Additionally, the rearing process used black cotton cloth for egg-bearing and light exclusion.

A phytotron (QingYu^®^, China) from Ningbo Qingyu Information Technology Co., Ltd. (Ningbo, China) was used to maintain a controlled environment for rearing *P. striolata*, allowing adjustments in temperature, humidity, and photoperiod. A binocular stereomicroscope (SZM-42, AOSVI^®^, Shenzhen, China) was utilized to observe individuals at various developmental stages and identify the sex of adults. Photographs of *P. striolata* at different developmental stages were captured using an ultra-depth-of-field 3D microscopic system (VHX-6000, KETENCE (China), Co., Ltd., Shanghai, China). The scale bars in the images represent 100 μm. A hole puncher, a device used to create uniform circular cuts, was employed to produce round pieces of radish root and cloth for the rearing of *P. striolata*. This tool allowed for the standardized preparation of dietary substrates and environmental materials, ensuring consistency in the experimental setup. A fine paintbrush was utilized for the collection of eggs and larvae. An autoclave was used for the sterilization of rearing material and equipment. Two devices were developed for the laboratory rearing of *P. striolata*. A suction trap device, designed for the adult collection, consisting of an insect storage tube (a 50 mL centrifuge tube), an entrance conduit (consisting of a 20 cm rubber tube and a plastic entry port), and a negative pressure conduit (comprising a 30 cm rubber tube and a gas suction nozzle) (Figure 1B). A 400-mesh gauze was used to cover the protruding air inlet in the storage tube to prevent insects from being sucked into the negative pressure conduit. A rearing device for immature *P. striolata*, consisting of a 24-well culture plate and its contents, was developed as well (Figure 1C–E). To maintain humidity within the rearing wells and reduce the infection of *P. striolata* and its food—radish roots—by pathogens, we designed a specialized substrate. One milliliter of this substrate, composed of water, agar, potassium sorbate, benzylpenicillin sodium, and pimaricin, was dispensed into each well of the plate and allowed to spread horizontally through slight vibration. The agar concentration was 2.5%, with the ratio of the other three components being 1000:100:10:1. Black or dark brown food-grade pigments were added to the substrate to enhance the visibility of immature individuals. Once the substrate solidified, a round piece of black cotton cloth was placed on its surface with an equal diameter of 16 mm matching the well. Round slices of radish root, measuring 10 mm in diameter and 3.5 mm in thickness, were placed at the center of the black round cloth to serve as larval food. After covering the wells with double layers of black cotton cloth, the plate was sealed with its lid. Ventilation holes with a diameter of 2 mm were punched into the lid at positions corresponding to each well. A schematic diagram for the rearing device of immature *P. striolata* is shown in Figure 1.

#### 2.2.2. The Laboratory-Rearing Process of *P. striolata*

In this study, a complete generation of *P. striolata*, encompassing the egg, larva, prepupa, pupa, and adult stages, was successfully reared using a method implemented within a phytotron maintained at 26 ± 1 °C and 70 ± 10% relative humidity, with a photoperiod of 12 h of light and 12 h of darkness.

The entire *B. rapa* plant with 8–9 true leaves underwent a thorough washing process, including the roots. Subsequently, roots were sequentially wrapped in moist absorbent cotton and a black cloth strip to ensure a consistent water supply and to accommodate adult oviposition. After positioning the plant upright in a transparent feeding bottle, a similar number of female and male adults, ranging from 30 to 50 individuals of each sex, were introduced into the container (Figure 1A). The adults feed on the leaves of the plant and lay their eggs on the black cloth, particularly along the edges or in the interstices between the layers of cloth.

Under a stereomicroscope, five eggs were transferred from the oviposition substrate to the interior rim of each rearing well containing a slice of radish using a fine paintbrush (Figure 1C).

A round slice of radish root, intended as food for the larvae, was placed beside the eggs in the culture well (Figure 1C). The culture well was subsequently covered with two layers of black cotton cloth, and the lid was secured in place (Figure 1D,E). Upon hatching, the 1st instar instinctively sought out and fed on the radish root, progressing through distinct developmental stages: larvae, prepupae, and pupae. The pupae eventually emerged as adults within the culture wells. Individuals at every stage can be observed and collected from the device throughout the rearing process.

After eclosion, adults were efficiently collected by a self-designed suction trap. Due to the distinct morphological differences in their antennae, the adults were sexed under a stereomicroscope, with the male’s fifth antennal segment being noticeably swollen compared to that of the female. Following this identification, the adults were transferred into a feeding bottle to facilitate reproduction for the next generation.

Life-history parameters were obtained for five F1 populations (LF, SH, NJ10, NJ11, NJP) and three consecutive generations (NJF1, NJF2, NJF3) of *P. striolata*. The developmental duration of the egg, larva, prepupa, and pupa stages of *P. striolata* was recorded in days, and the recorded individuals were sourced from the same batch but different oviposition substrates (Table S1). The duration recording began on each individual’s first day of the egg stage, and each individual of *P. striolata* was considered a biological replicate. The survival rate at each developmental stage was calculated by dividing the number of individuals developing into the next stage by the initial number of individuals (Table S2). For a population, eggs from a batch were grouped according to their oviposition substrate, with survival data documented across the egg, larva, prepupa, and pupa stages, and each group of *P. striolata* was considered a biological replicate. The female ratio, length of the hind tibia (measured with the unit “μm”), and fluctuating asymmetry (FA, measured with the unit “μm”) of adults in the NJ10, NJ11, and SH populations were recorded (Table S3). The length of the hind tibia was calculated using the mean value of the left and right hind tibiae. The FA was determined from the absolute differences in length between the two hind tibiae.

### 2.3. Statistical Analyses

The binary data, including the hatchability of the egg, the survival rate of the larva and prepupa, the eclosion rate of pupa, and the female ratio as influenced by different populations and generations, were analyzed using a generalized linear model (GLM) with a quasi-binomial distribution (using a logit link function). GLMs with a quasi-Poisson distribution (using a log link function) were used to analyze the developmental durations of egg, larva, prepupa, and pupa as affected by various populations and generations. The effects of different populations on the length of the hind tibiae and fluctuating asymmetry (FA) of adults were estimated using GLM with a Gaussian distribution (using an identity link function). The homogeneities of the GLMs were tested using the Breusch–Pagan tests and Shapiro tests [41,42]. Analyses were carried out with R ver. 4.0.1 [43]. Based on the models identified, car::Anova(type = II tests) was used to look for significant model terms. To assess differences between pairs of means, post hoc pairwise comparisons were conducted using the Tukey–Kramer test based on the emmeans::emmeans (model, pairwise~significant factor, type = “response”).

## 3. Results

### 3.1. Phyllotreta striolata: A Comprehensive View of Developmental Stages Through Laboratory-Rearing

**Figure 2:**
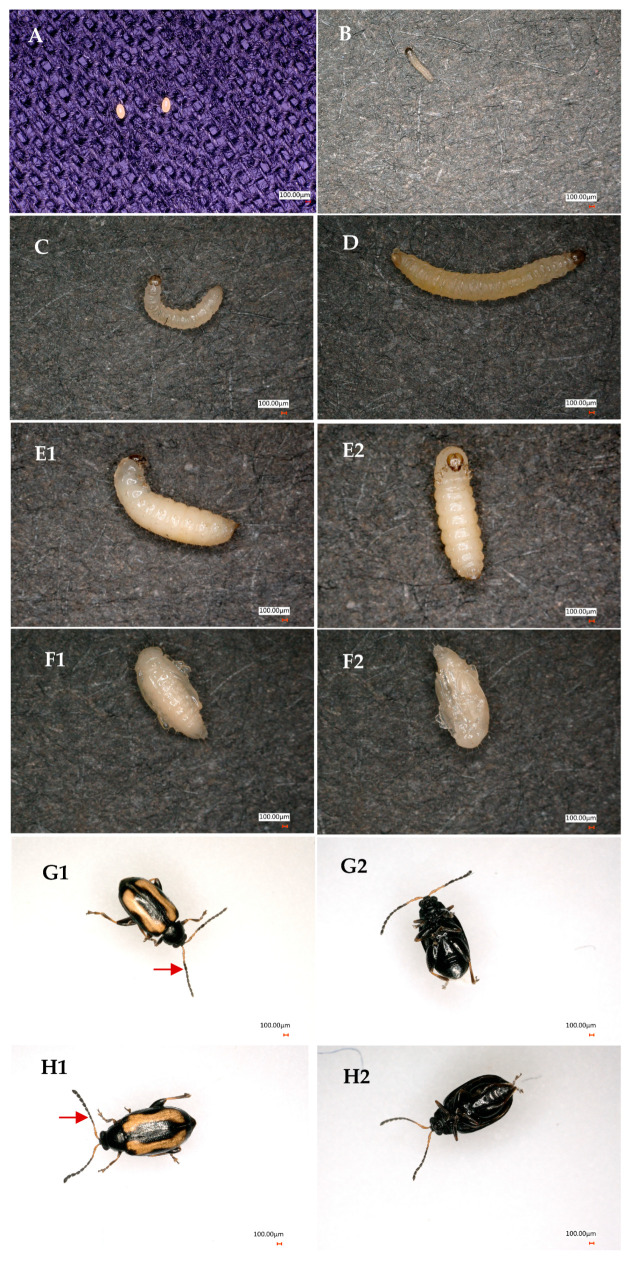
The *Phyllotreta striolata* at various developmental stages acquired by the laboratory-rearing method. (**A**) Egg, (**B**) first instar, (**C**) second instar, (**D**) third instar, (**E1**) prepupa in side view, (**E2**) prepupa in ventral view, (**F1**) pupa in dorsal view, (**F2**) pupa in ventral view, (**G1**) male adult in dorsal view (with an enlarged fifth antennal segment indicated by a red arrow), (**G2**) male adult in ventral view, (**H1**) female adult in dorsal view (with a normal fifth antennal segment indicated by a red arrow), (**H2**) female adult in ventral view. The scale bar in the figures represents 100 μm.

A complete generation of *P. striolata* has been successfully reared in the laboratory using the method delineated in the Section 2. Individuals at different developmental stages are illustrated in Figure 2.

### 3.2. The Developmental Durations and Survival Rates of P. striolata in Five Laboratory-Rearing F1 Populations

#### 3.2.1. The Developmental Durations of *P. striolata* in Five F1 Populations

The developmental durations of *P. striolata* across four stages (egg, larva, prepupa, and pupa) in five F1 populations (NJ10, NJ11, NJP, SH, LF) were acquired by the laboratory-rearing method.

**Figure 3:**
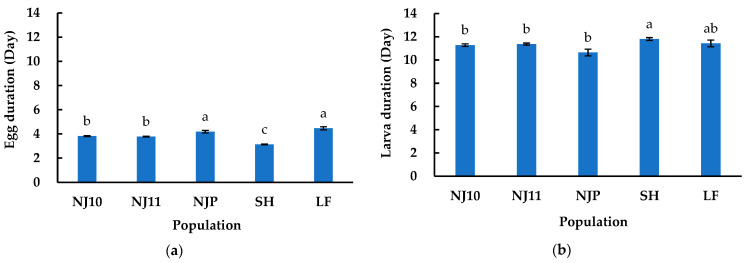

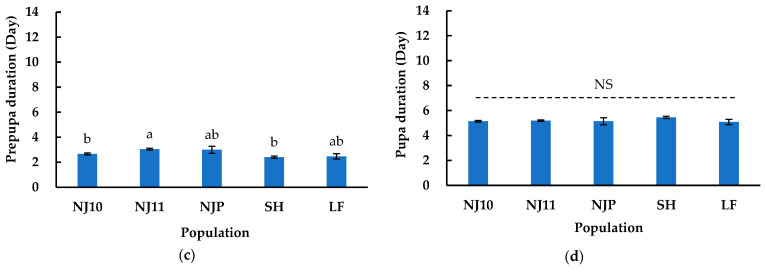
The developmental durations of *Phyllotreta striolata* across four developmental stages in five F1 populations. (**a**) Egg durations in five populations; (**b**) larva durations in five populations; (**c**) prepupa durations in five populations; (**d**) pupa durations in five populations. The bars indicate standard errors. The same letters at the top of the columns indicate no significant difference in the parameter between them (*p* > 0.05). NS above the dotted line indicates no significant difference in the parameter among all populations (*p* > 0.05).

Egg duration was significantly influenced by different populations (*F_4, 3115_* = 107.120, *p* < 0.001). The SH population (mean ± SE, 3.13 ± 0.03 days, *n* = 1181) exhibited a significantly shorter egg duration than LF (4.47 ± 0.13 days, *n* = 74) (*z* = 11.765, *p* < 0.001) (Figure 3a). There was no significant difference in egg duration between NJ10 (3.82 ± 0.04 days, *n* = 831) and NJ11 (3.78 ± 0.03 days, *n* = 918) (*z* = 0.663, *p* = 0.964), nor between NJP (4.19 ± 0.10 days, *n* = 116) and LF (*z* = 1.734, *p* = 0.413) (Figure 3a).

Significant variation was observed in the larval duration among five populations (*F_4, 803_* = 4.65, *p* = 0.001). The SH population (mean ± SE, 11.81 ± 0.12 days, *n* = 198) exhibited a significantly longer larval duration than that of NJ10 (11.28 ± 0.11 days, *n* = 220) (*z* = 3.149, *p* = 0.014), NJ11 (11.37 ± 0.09 days, *n* = 324) (*z* = 2.864, *p* = 0.034), and NJP (10.65 ± 0.29 days, *n* = 31) (*z* = 3.510, *p* = 0.0041), but it was not true when compared with the LF population (11.43 ± 0.29 days, *n* = 35) (*z* = 1.202, *p* = 0.750) (Figure 3b). No statistical differences in larval duration were found among NJ10, NJ11, NJP, and LF populations, nor between LF and SH (*p* > 0.05) (Figure 3b).

Prepupa durations varied significantly among five populations (*F_4, 517_* = 9.240, *p* < 0.001) (Figure 3c). The prepupa duration of NJ11 (mean ± SE, 3.05 ± 0.07 days, *n* = 220) was significantly longer than that of NJ10 (2.66 ± 0.08 days, *n* = 160) (*z* = 3.686, *p* = 0.0021) and SH (2.41 ± 0.09 days, *n* = 110) (*z* = 5.489, *p* < 0.001), but it was not true when compared with NJP (3.00 ± 0.28 days, *n* = 13) (*z* = 0.155, *p* = 0.999) and LF (2.47 ± 0.21 days, *n* = 19) (*z* = 2.342, *p* = 0.132). No significant differences were observed among NJ10, NJP, SH, and LF (*p* > 0.05) (Figure 3c).

Pupa duration was not significantly influenced by different populations (*F_4, 381_* = 2.160, *p* = 0.073) (Figure 3d). The pupa durations (mean ± SE) of NJ10, NJ11, NJP, SH, and LF were 5.14 ± 0.07 days (*n* = 114), 5.19 ± 0.06 days (*n* = 174), 5.14 ± 0.29 days (*n* = 7), 5.45 ± 0.09 days (*n* = 78), and 5.08 ± 0.21 days (*n* = 13), respectively (Figure 3d).

#### 3.2.2. The Survival Rates of *P. striolata* in Five F1 Populations

The survival rates of *P. striolata* across four developmental stages (egg, larva, prepupa, and pupa) in five F1 populations were obtained using the laboratory-rearing method.

**Figure 4:**
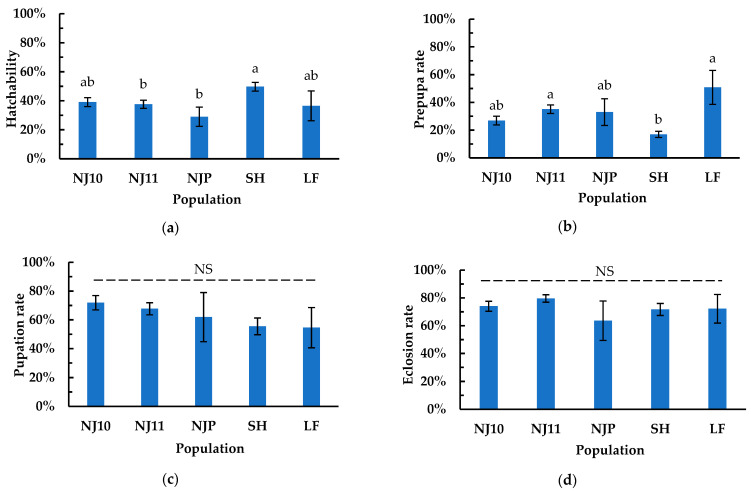
The survival rates of *Phyllotreta striolata* across four developmental stages in five F1 populations. (**a**) Egg hatchabilities in five populations; (**b**) prepupa rates of larvae in five populations; (**c**) pupation rates in five populations; (**d**) eclosion rates in five populations. The bars indicate standard errors. The same letters at the top of the columns indicate no significant difference in the parameter between them (*p* > 0.05). NS above the dotted line indicates no significant difference in the parameter between all populations (*p* > 0.05).

Egg hatchability was significantly influenced by different populations (*F_4, 51_* = 3.383, *p* = 0.016). SH population (mean ± SE, 49.71% ± 2.98%, *n* = 9) exhibited significantly higher hatchability than that of NJ11 (37.60% ± 2.80%, *n* = 10) (*z* = 2.933, *p =* 0.028) and NJP (29.02% ± 6.63%, *n* = 11) (*z* = 2.572, *p =* 0.046), but it was not true when compared with NJ10 (39.13% ± 3.05%, *n* = 22) (*z* = 2.458, *p* = 0.101) and LF population (36.52% ± 10.26%, *n* = 4) (*z* = 1.181, *p* = 0.762) (Figure 4a). No significant differences were observed among the LF, NJ10, NJ11, and NJP populations (*p* > 0.05) (Figure 4a).

The prepupa rate exhibited significant variation among the five populations (*F_4, 47_* = 7.021, *p* < 0.001). The SH population (mean ± SE, 16.92% ± 2.21%, *n* = 9) had a significantly lower prepupa rate than that of NJ11 (35.05% ± 3.13%, *n* = 10) (*z* = 4.672, *p* < 0.001) and LF (50.77% ± 12.27%, *n* = 4) (*z* = 3.148, *p* = 0.014), but it was not true when compared with NJ10 (26.89% ± 3.14%, *n* = 20) (*z* = 2.640, *p* = 0.063) and NJP (32.98% ± 9.59%, *n* = 9) (z = 1.912, *p* = 0.311) (Figure 4b). No statistically significant differences in prepupa rate were observed among NJ10, NJ11, NJP, and LF (*p* > 0.05) (Figure 4b).

The pupa rate was not significantly influenced by the different populations (*F_4, 44_* = 1.390, *p =* 0.253) (Figure 4c). The pupa rates (mean ± SE) of NJ10, NJ11, NJP, SH, and LF were 71.90% ± 5.02% (*n* = 20), 67.71% ± 4.23% (*n* = 10), 61.90% ± 17.14% (*n* = 6), 55.50% ± 5.82% (*n* = 9), and 54.55% ± 14.02% (*n* = 4), respectively (Figure 4c).

The eclosion rate was not significantly influenced by different populations (*F_4, 39_* = 1.025, *p =* 0.407) (Figure 4d). The eclosion rates (mean ± SE) of NJ10, NJ11, NJP, SH, and LF were 73.97% ± 3.55% (*n* = 17), 79.63% ± 2.68% (*n* = 10), 63.64% ± 14.18% (*n* = 4), 71.70% ± 4.28% (*n* = 9), and 72.22% ± 10.32% (*n* = 4), respectively (Figure 4d).

### 3.3. The Developmental Durations and Survival Rates of P. striolata in Three Laboratory-Rearing Consecutive Generations

Three consecutive generations of *P. striolata* from the NJ population, namely NJF1, NJF2, and NJF3, were obtained using a novel laboratory-rearing method in this study. The analysis of life-history parameters concentrated on the durations and survival rates of *P. striolata* as it progressed through four developmental stages across three populations (Table 2).

Egg duration was significantly influenced by different generations (*F_2, 424_* = 160.780, *p* < 0.001) (Table 2). NJF1 exhibited a significantly longer egg duration than that of NJF2 (*z* = 11.800, *p* < 0.001) and NJF3 (*z* = 16.197, *p* < 0.001). Significant differences were observed between NJF2 and NJF3 (*z* = 4.011, *p* < 0.001). In contrast, larva duration was not significantly influenced by the different generations (*F_2, 163_* = 0.185, *p* = 0.832) (Table 2). Prepupa duration varied significantly among the three generations (*F_2, 115_* = 7.590, *p* < 0.001). NJF2 exhibited a significantly longer prepupa duration than NJF1 (*z* = 3.815, *p* < 0.001), but no significant differences were found between NJF3 and both NJF1 (z = 1.999, *p* = 0.112) and NJF2 (*z* = 1.520, *p* = 0.281) (Table 2). Pupa duration also exhibited significant variation among the three generations (*F_2, 72_* = 4.350, *p* = 0.016). NJF1 had a significantly longer pupa duration than that of NJF2 (*z* = 2.878, *p* = 0.011), but no statistically significant differences were observed between NJF3 and both NJF1 (*z* = 0.210, *p* = 0.976) and NJF2 (*z* = 2.296, *p* = 0.056).

Egg hatchability was not significantly influenced by different generations (*F_2, 9_* = 3.441, *p* = 0.078). NJF1 exhibited significantly higher hatchability than NJF2 (*z* = 2.494, *p* = 0.034), but no significant differences were observed between NJF3 and both NJF1 (*z* = 1.900, *p* = 0.139) and NJF2 (*z* = 0.685, *p* = 0.772) (Table 2). The prepupa rates (survival rate of larvae) varied significantly among the three generations (*F_2, 9_* = 5.797, *p* = 0.024). The prepupa rate of NJF1 was significantly higher than that of NJF2 (*z* = 3.035, *p* = 0.0068) and NJF3 (*z* = 2.404, *p* = 0.043) (Table 2). No significant difference was observed between NJF2 and NJF3 (*z* = 0.875, *p* = 0.656). The pupation rate (survival rate of prepupae) was significantly affected by different generations (*F_2, 8_* = 4.594, *p* = 0.047). A significant difference in pupation rate was found between NJF1 and NJF3 (*z* = 2.909, *p* = 0.010), while non-significant differences were observed between NJF2 and both NJF1 (*z* = 1.586, *p* = 0.252) and NJF3 (*z* = 0.741, *p* = 0.739) (Table 2). The eclosion rate was not significantly influenced by the different generations (*F_2, 8_* = 0.768, *p* = 0.496) (Table 2).

### 3.4. The Three Response Parameters in Laboratory-Rearing F1 P. striolata Populations

**Figure 5:**
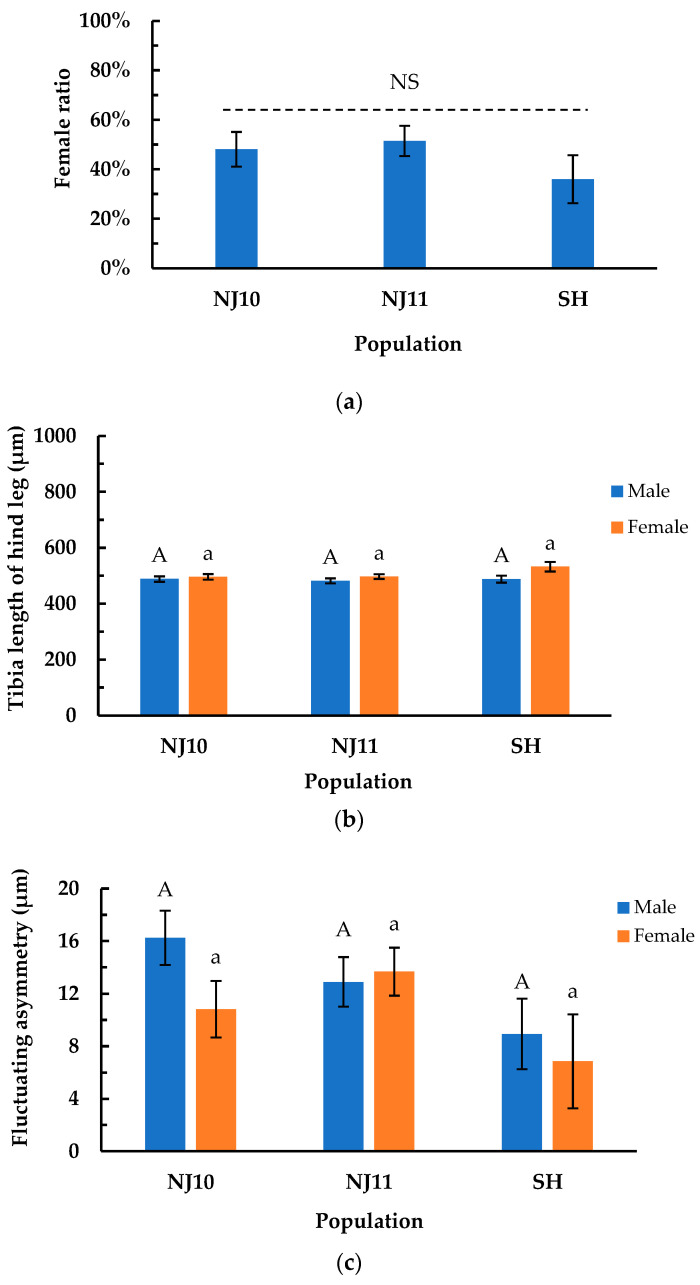
The three response parameters in laboratory-rearing F1 *P. striolata* populations. (**a**) Female ratios in three populations; (**b**) tibia length of hind leg of *P. striolata* in three populations; (**c**) fluctuating asymmetry of *P. striolata* in three populations. Fluctuating asymmetry was obtained based on the absolute differences in length between the two tibiae of the hind legs of *P. striolata*. The bars indicate standard errors. Columns with the same uppercase (for males) or lowercase (for females) letters at the top indicate no significant difference in the parameter between their corresponding populations (*p* > 0.05). NS above the dotted line indicates no significant difference in the parameter between all populations (*p* > 0.05).

The female ratio, length of the hind tibia, and fluctuating asymmetry of *P. striolata* from NJ10, NJ11, and SH populations were obtained through laboratory rearing and measuring. The female ratio was not significantly influenced by the different populations (*F_2, 142_* = 0.870, *p* = 0.420) (Figure 5a). The female ratios (mean ± SE) of NJ10, NJ11, and SH populations were 48.08% ± 7.00% (*n* = 52), 51.47% ± 6.12% (*n* = 68), and 36.00% ± 9.70% (*n* = 25), respectively.

The hind tibia lengths (mean ± SE) of *P. striolata* in NJ10, NJ11, and SH were 492.18 ± 7.00 μm, 489.39 ± 6.12 μm, and 510.03 ± 10.51 μm. The hind tibia length was not significantly influenced by population (*F_2, 139_* = 1.030, *p* = 0.361). No significant variation in hind tibia length among males was observed across the three populations, as evidenced by the following comparisons: NJ10 vs. NJ11 (*z* = 0.501, *p* = 0.871), NJ10 vs. SH (*z* = 0.026, *p* = 0.100), and NJ11 vs. SH (*z* = 0.400, *p* = 0.916) (Figure 5b). Similarly, the hind tibia length of females did not exhibit significant variation among the three populations (NJ10 vs. NJ11: *z* = 0.074, *p* = 0.997; NJ10 vs. SH: *z* = 1.840, *p* = 0.157; NJ11 vs. SH: *z* = 1.862, *p* = 0.150). However, hind tibia length was significantly affected by sex, with females exhibiting a significantly longer hind tibia than males (*z* = 2.385, *p* = 0.017).

The fluctuating asymmetries (FAs) (mean ± SE) derived from the hind tibia length data of the NJ10, NJ11, and SH populations were 13.538 ± 1.495 μm, 13.290 ± 1.307 μm, and 7.898 ± 2.244 μm, respectively. FA was not significantly influenced by population (*F_2, 139_* = 2.710, *p* = 0.070). Males exhibited no significant variation in FA among the three populations (NJ10 vs. NJ11: *z* = 1.197, *p* = 0.455; NJ10 vs. SH: *z* = 2.152, *p* = 0.080; NJ11 vs. SH: *z* = 1.209, *p* = 0.448) (Figure 5c). Similarly, for females, there were no significant differences in FA among the three populations (NJ10 vs. NJ11: *z* = 1.010, *p* = 0.570; NJ10 vs. SH: *z* = 0.947, *p* = 0.610; NJ11 vs. SH: *z* = 1.693, *p* = 0.208) (Figure 5c). Additionally, no significant difference in FA between males and females was observed among the three populations (*z* = 1.122, *p* = 0.262).

## 4. Discussion

### 4.1. The Analysis of Life-History Parameters of P. striolata

The life-history parameters of a population are important for life table research, which necessitates the collection and maintenance of comprehensive data records, including developmental durations, survival rates, and other traits for each individual at all stages from birth to death [24,25]. Life tables form the foundation of insect ecological studies, serving as a primary research tool for the analysis of population stage structure and interactions between populations, aiding in the estimation of population dynamics, and playing a significant role in developing effective pest management strategies [24,25,44]. The survival rates and developmental durations of *P. striolata* on Brassicaceae plants constitute the basis for predicting occurrence periods, damage levels, and generation numbers. Calculating the adult sex ratio can aid in predicting population dynamics [24,27]. As a significant biomarker, fluctuating asymmetry (FA) provides insights into insect adaptations and the effects of environmental pressures [28]. Typically, FA is calculated as the absolute mean of the differences between the right and left sides of a given trait, such as wing length, centroid, and thorax [45,46]. Given that the hind legs of *P. striolata* enhance the efficiency of food searching and predator evasion, which are crucial for its field movement and dispersal, this study calculates FA based on the length of the hind tibia. The life-history parameters of insects are influenced by their geographical origin [29,30,31,32]. Studies have revealed that, under the same laboratory-rearing conditions, different geographical populations of *S. avenae*, *P. xylostella*, *R. padi* from China and *M. domestica* from Pakistan exhibit significant differences in immature development duration and mortality, generation time, adult fecundity, and lifespan [29,30,31,32]. Researchers attribute these differences to genetic adaptations of populations in different environments and the long-term effects of local environmental conditions. In this study, five populations from three different provinces in China were collected and reared indoors to obtain parameters such as survival rates, development periods, female sex ratio, and FA. The laboratory-rearing method was validated, and the differences in life-history traits among populations of different geographical origins provided references for developing management strategies for *P. striolata* in various regions. Additionally, one of the geographical populations was reared indoors for three consecutive generations. By comparing differences in life-history parameters across these generations, the reliability of the rearing method was further verified, and a foundation was laid for further research into the causes of differences among geographical populations.

The developmental durations of *P. striolata* across four life stages were recorded. Statistical analysis revealed no significant differences in pupal duration among five F1 populations or in larval duration across the three consecutive generations, and other stages exhibited significant variation between populations (Figure 3d; Table 2). In the five F1 populations, for those originating from the same host, *Brassica rapa var. chinensis*, but with different geographical origins (NJ11, LF, SH), the SH population had a significantly shorter egg duration and a longer larval duration compared to other populations, with the latter being significantly different from NJ11. The NJ11 and LF populations showed no significant differences except in egg duration. For the three populations of the same geographical origin (NJ10, NJ11, NJP), there were no significant differences in *P. striolata* populations originating from *Brassica rapa* and *Raphanus sativus*, except for in egg duration. In the three consecutive generations of *P. striolata*, excluding egg duration, the other three stages showed no significant differences between NJF3 and the first two generations. This may indicate that, during the laboratory breeding across generations, these three stages exhibited a stronger convergence in adaptation to the rearing environment we provided. In this study, the average duration for the egg, larva, prepupa, and pupa stages across the five F1 populations were 3.88, 11.31, 2.72, and 5.20 days, respectively, while in the three consecutive generations, these averages were 4.05, 11.22, 2.92, and 5.08 days, respectively. Zhang et al. reported that *P. striolata* has a four-stage life cycle in the autumn generation on *B. campestris* in the field: eggs lasting 6 days, larvae and prepupae together lasting 18 days, and pupae lasting 9 days [1]. The reduced durations observed across the three life stages of *P. striolata* in our study may be attributed to the varying conditions between laboratory and field environments.

All survival data for *P. striolata* were meticulously recorded, tracking individuals from various egg oviposition substrates throughout their developmental process (Table S2). This approach provided valuable insights into the developmental dynamics achieved through our rearing method. For survival rates across all four stages, at least four F1 populations showed no statistically significant differences from one another (Figure 4). In the five F1 populations, three populations originating from different regions showed that the SH had a lower larval survival rate compared to the NJ11 and LF populations, with no significant differences observed in other developmental stages. For the three populations from Nanjing City (NJ10, NJ11, NJP), there were no significant differences in survival rates at their different stages, although they derived from different hosts. The average survival rates across the four developmental stages for NJF1 were higher than those for NJF2 and NJF3, with no significant differences observed between the latter two, possibly due to the initial three generations still adapting to the artificial environment. The average survival rates across the egg, larval, prepupal, and pupal stages for the five F1 populations were 38.40%, 32.52%, 62.31%, and 72.23%, respectively. The corresponding rates for the three successive generations were 37.94%, 42.28%, 66.02%, and 59.24%. These two data sets indicate that, under the rearing conditions of this study, the survival rates of eggs and larvae are relatively low, while those of prepupae and pupae are comparatively high (Figure 4; Table 2). From these results, it can be inferred that during the developmental process of *P. striolata*, the early stages exhibit limited adaptability to the rearing conditions. In contrast, *P. striolata* enters a defensive physiological state in the later stages, which confers resistance to factors potentially hindering growth.

To gain deeper insights into the adaptability of *P. striolata* to the rearing conditions of the method, we measured three parameters: female ratio, hind tibia length, and FA in three F1 populations (NJ10, NJ11, SH). The female ratio showed no significant variation among the three groups (Figure 5a). When transformed into a sex ratio (female/male), the proportions for the NJ10 and NJ11 populations were 0.93:1.00 and 1.06:1.00, respectively, approximating a 1:1 ratio. For the SH population, the ratio is 1.00:1.78. For the first two populations, the rearing conditions did not induce a sexual imbalance, but this was not the case for the SH population. If this situation is not a coincidence, the rearing method requires further optimization. The hind tibia length exhibited no significant differences among the three F1 populations for either sex (Figure 5b), but the females had a significantly longer hind tibia than males (*p* < 0.05), which may be associated with sexual dimorphism. FA was not influenced by population or sex among the three groups (Figure 5c). Since FA can reflect the impact of environmental stress on individual development [28], this finding potentially indicates three F1 populations did not show significant differences in adaptability under the laboratory-rearing conditions.

Geographic isolation of insect populations may facilitate the accumulation of mutations in different environments, potentially leading to the formation of allopatric species [47]. The accumulation of these mutations is likely a result of the combined effects of intrinsic species characteristics and external environmental factors [30]. Investigating life-history parameters across different geographic populations can offer insights into the causes of this phenomenon. In this study, the lack of significant differences in some life-history parameters among various geographic populations can be attributed to two possible explanations. First, the genetic basis influencing these parameters may remain relatively stable when environmental conditions change, such as transitioning to laboratory breeding conditions. Second, these parameters may not be sensitive to genetic differences when compared to current environmental pressures. As for the differences in life-history parameters among geographic populations, we believe that the data provided in this study are not sufficient to clarify the underlying causes. Research into the plasticity and genetic basis of key life-history traits in insects is still limited. Generally, comparing different geographic populations and identifying variations is not enough to conclude that life-history trait variations are explained by genetic differentiation [30]. Therefore, further exploration is needed to understand the relationships between insect life-history traits and their geographic origins.

### 4.2. The Rearing Characteristics of P. striolata at Different Developmental Stages

In this study, we developed a laboratory-rearing method for *P. striolata* leveraging the habitat, behavior, and developmental characteristics. Based on the analysis of the life table parameters, this method has been proven to be effective and reliable. During the adult rearing process, the plant’s roots were sequentially wrapped in moist cotton and multiple layers of black cloth to ensure a consistent water supply and facilitate oviposition (Figure 1A). With this approach, an entire plant of *B. rapa* can survive in the rearing bottle for at least seven days, eliminating the need for frequent replacements. We developed a novel device to rear immature *P. striolata* (Figure 1C–E). The moisturizing substrate in the wells of the culture plate prevents microbial infection, allowing radish slices to remain fresh throughout the immature phase of *P. striolata*, eliminating the need to replace the slices or transfer individuals to new food sources, thereby reducing the risk of physical harm and pathogen infection to *P. striolata*. During the rearing process, the leaves of *B. rapa* and the roots of *Raphanus sativus* were utilized as food sources for adults and immature individuals, respectively. For the adults, two primary reasons can account for the choice of feed. First, according to the findings of Liu et al. [11], *P. striolata* exhibits a stronger preference for the true leaves of *B. rapa* compared to radish. Second, *B. rapa* with 8–9 true leaves has a smaller root volume, which facilitates wrapping with the water-supplying cotton and oviposition substrate. Furthermore, considering the impact of wax on insect feeding preferences, we selected a variety of *B. rapa* with thin wax layers. In two previous studies, eggs were laid on host plant leaves or seedlings within feeding containers [37,48], yet other reports indicate that *P. striolata* prefers the rhizosphere [3,9,12]. In this study, we did not observe any eggs on the surface of the leaves. Regarding larval feed, Zhang et al. found that the survival rate of larvae reared on radish taproots was higher than that of those fed on radish seedlings, as well as higher than that of larvae feeding on seedlings of *Brassica rapa* var. *chinensis*, *Brassica juncea* var. *foliosa*, and *Brassica campestris* var. *pekinensis* [40]. Furthermore, Zhang et al. [40] noted that the eclosion rate of *P. striolata* feeding on radish seedlings was higher compared to those feeding on the other three vegetables, suggesting a preference for radish roots. The roots of *Raphanus sativus* var. *longipinnatus*, characterized by their green skin and interior, provide a high contrast against the larvae, facilitating easy collection, and serve as a convenient food source when sliced into rounds. In a previous study, newly hatched larvae were transferred to Petri dishes lined with 3 to 5 layers of leaves for development until eclosion [37], although other studies indicate that larvae may prefer feeding on the roots of Brassica plants [3,9,12,40]. The application of preservatives and antibiotics in this study can reduce pathogen infections in *P. striolata* and radishes. Additionally, newly emerged adults exhibit a white body color and a soft body wall, which should be collected after their color changes to black and the central band of the elytra turns yellow. In this study, we established an effective laboratory-rearing method for *P. striolata* to provide tested insects at all developmental stages and assist in maintaining indoor populations. Previous studies have reported rearing methods for different growth stages of *P. striolata*, which will be discussed in the following text.

For the rearing of adults and the collection of eggs, several studies have demonstrated varying characteristics. In the study conducted by Xian et al. [35], seedlings were placed in a rearing bottle lined with black cloth, where eggs were laid on the surface of the fabric. Due to the lack of a water supply, the seedlings need to be replaced promptly. During our rearing process, the interlayer space created by wrapping cloth strips provides an oviposition site, allowing the host plant in the rearing bottle to remain fresh for up to seven days without replacement, due to the continuous water supply from the moist cotton. Nagalingam and Costamagna developed three methods for adult rearing [36]. For the pre-hibernation rearing of adults, glass jars lined with peat moss at the base were used, with Napa cabbage leaves as food [36]. For the mass collection of adults, parent adults were reared in cages containing pots of canola [36]. To collect eggs, an oviposition tub with holes at the bottom, wrapped in cheesecloth, was placed on a layer of paper towels covering the soil, where the adults fed on a cabbage leaf and laid eggs on the paper towels through the cloth [36]. Our study aimed to achieve generational rearing and to collect individuals of *P. striolata* at various developmental stages, which differed from previous methods. In their research, the authors identified pest contamination as a significant concern [36]. They noted that fungal infections in the oviposition tubs could potentially impact adult survival and reduce egg hatchability. The authors attributed the increased humidity in the oviposition tubs, which heightens the risk of fungal invasion, to the presence of cabbage leaves. To mitigate this issue in our study, we incorporated ventilation holes in the lids of the adult-rearing bottles. Additionally, during the rearing and oviposition of adult *P. striolata*, 30 to 50 pairs were placed in breeding bottles, a number established through trials. Insect density significantly impacts reproduction [49,50,51,52,53]. For *Tenebrio molitor*, egg production increases with adult density within a certain density range, but overcrowding reduces fecundity, decreasing individual female egg production and increasing adult mortality [49,50]. The former study suggested an optimal rearing density of 0.25 ind/cm^2^ [49], and the latter indicated 0.08 ind/cm^2^ [50]. Similarly, *Tribolium castaneum* shows decreased individual egg production and offspring maturation rate with higher adult density [51]. This pattern is also observed in other Diptera and Lepidoptera species [52,53]. *P. striolata* has strong mobility, is capable of flying and jumping, and exhibits gregarious feeding behavior [12,13,22]. Based on its ecological habits, we determined a population density of 0.07–0.12 ind/cm^3^, which ensures egg production without frequent feed replacement. Below this range, total egg production drops; above it, the host plant supply cannot be maintained for seven days.

Various methods have been developed to preserve eggs and rear larvae. In the study by Wang et al. [34], collected eggs were placed on a moist cloth within a Petri dish lined with filter paper. This method presents challenges in controlling humidity inside the Petri dish, as moisture readily evaporates and condenses into water droplets on the lid, resulting in a drier egg carrier and an increased risk of mold contamination. Our device incorporates an agar substrate with antibiotics and a lid with ventilation holes to mitigate these issues. Liu et al. [33,54] placed first-instar larvae on the surface of plant roots, subsequently wrapping them in filter paper and cotton cloth. In other research, larvae were also reared on radish roots in Petri dishes, within radish grooves, or on seedlings in sandy soil [34,35,48], with radishes or larvae being replaced or transferred as needed. These methods heighten the risks of harming or losing larvae and introducing pathogens. In this study, the provision of food in the rearing wells eliminated the need for radish replacement and insect removal, thereby facilitating observation and acquisition. At 26–30 °C, the study of Xian et al., using a multilayer radish slice method, achieved a prepupal rate of 34.3% [35]. Our study observed prepupal rates ranging from 16.92% to 50.77% across five F1 populations and from 27.38% to 62.41% across three generations at 26 ± 1 °C. Possibly, the growing environment and temperature are factors influencing survival rates. In their study, first-instar larvae took 8.5 to 16.0 days to develop into pupae [35], while in this study, the duration was 10.65 to 11.81 days for five populations and 11.13 to 11.38 days for three generations. The results observed in both studies suggest that differences in rearing methods may have a minimal effect on larval duration. In the research conducted by Nagalingam and Costamagna [36], eggs from oviposition tubs were placed on canola seedlings in pots and enclosed in mesh cages, with regular observations made to collect the emerged adults. While their method can maintain a laboratory strain, it may not be suitable for observing and collecting *P. striolata* at various developmental stages, which is not challenging by our approach. Furthermore, the antimicrobial foundation of our rearing method prevents microbial growth, which is necessary even when using soil in pots, as in their method. In addition, Nagalingam and Costamagna suggested that removing flea beetle adults from host plant cages could increase the risk of contamination by aphids and diamondback moths, and limiting the collection of fresh adults could lead to generation overlap, complicating the precise analysis of eclosion rates, as evidenced by the 138% eclosion rate they reported [36]. In our rearing process, *B. rapa* is cultivated in a pest-free greenhouse that is subjected to ultraviolet light exposure and pesticide applications before sowing. Additionally, strict sterilization and pest eradication measures are implemented for seeds, pots, soil, and cultivation tools. Newly emerged adults can be promptly collected from the wells of the culture plates, thereby reducing the risk of the aforementioned issues. In the study by Nagalingam and Costamagna [36], methods involving canola pots placed in mesh cages for the bulk collection of adults or eggs and larvae (methods 2 and 3) facilitated the recording of *P. striolata*’s immature stage duration, which varied from 25 to 30 days or 26 to 33 days at 24 °C. In our study, the duration ranged from 22 to 24 days at 26 ± 1 °C (Figure 3; Table 2). Three potential explanations account for the observed differences. First, the provision of food and space in the rearing wells enhances foraging efficiency and reduces competition. Second, our larvae are protected from complex soil environments that could potentially impact their development. Lastly, the slightly elevated temperature in our study may contribute to accelerated development. In addition to recording the duration of developmental stages, the methods employed also captured the sex ratio of emerging adults, which was 1.1–1.7 females to 1.0 males [32]. In contrast, our study observed the sex ratios (female/male) were 0.93:1.00, 1.06:1.00, and 0.56:1.00. These discrepancies may be attributed to differences in rearing methods. As previously mentioned, Patricio and Ocampo used leaves of *B. rapa* to rear larvae in Petri dishes, recording several duration parameters of *P. striolata* [37]. The duration ranges for egg, larva, prepupa, and pupa were 3–5 days, 8–17 days, 1–3 days, and 3–6 days, respectively [37]. In our study, we obtained narrower ranges for larval duration ranges (Figure 3; Table 2). In our opinion, this difference may arise from the distinct food sources and rearing methods employed.

Several methods for rearing prepupal and pupal stages have been reported in previous studies. In the study by Xian et al. [35], late third-instar larvae were transferred to the host’s roots, covered with sand, wrapped in a damp cotton cloth, and placed in a Petri dish to collect pupae and adults. This method necessitates the transfer of larvae and does not allow for the stage-by-stage collection of insects, as demonstrated in our study. Additionally, they explored a rearing method using multi-layered radish slices [35]. In our study, the pupal period for eight populations averaged between 4.56 and 5.45 days at 26 ± 1 °C, which is consistent with the 4 to 6 days reported by Xian et al. at 26–30 °C when utilizing radish slices [35]. This similarity may be attributed to the special developmental stage. In a previous study, larvae were nurtured in radish grooves to facilitate pupation, after which the pupae were transferred to a damp cloth within a Petri dish for eclosion [34]. In our study, there is ample eclosion space in the culture wells, eliminating the need for transfer. With the successful rearing of *P. striolata* for three consecutive generations in the laboratory, our rearing method is anticipated to support the long-term maintenance of the laboratory population.

The life-history parameters of *P. striolata* obtained in this study can serve as a reference for its indoor rearing, as well as for physiological and toxicological research. These parameters contribute to the understanding of the ecology of *P. striolata*, facilitating the investigation into its relationships with host plants and natural enemies. Furthermore, they can form the basis for predicting field population dynamics and guiding pest control strategies. The laboratory-rearing method developed in this study is practical, providing insects at various developmental stages to meet diverse research needs, and it establishes a foundation for the long-term maintenance of specific strains. Additionally, this method offers a reference for the study of pests with similar ecological habits. Of course, this study has certain limitations, including the need to enhance the efficiency of egg collection methods and to improve hatching and larval survival rates. To address these issues, we are exploring a method that involves washing eggs from oviposition substrates, followed by rearing. Furthermore, we are developing semi-artificial diets for the rearing of immature individuals. Our study provides insects with consistent growth periods primarily through single-well rearing, which ensures stable growth environments. If the objective is solely to obtain a batch of adults, a direct contact method with the plant can be employed for the oviposition substrate that carries eggs [35,36].

## 5. Conclusions

In this study, we detailed a laboratory-rearing method for *P. striolata*. Using this method, we successfully established five F1 populations (LF, SH, NJP, NJ10, NJ11) and three consecutive generations (NJF1, NJF2, NJF3) derived from field parental populations in the laboratory. This enabled us to meticulously record life-history parameters across four developmental stages. Overall, for the five populations, the durations of the egg, larva, and prepupa stages, as well as the survival rates of eggs and larvae, were significantly influenced by the different populations, respectively. For three consecutive generations, the durations of eggs, prepupae, and pupae and the survival rates of larvae and prepupae were also significantly influenced by different generations, respectively. Notably, except for egg duration and larval survival rate, there were no significant differences in duration and survival rates between NJF3 and the first two generations. The populations did not significantly influence the female ratio, hind tibia length, and FA, indicating similar adaptability of the three F1 populations to the rearing conditions. This study presents a reliable laboratory-rearing method for *P. striolata* along with its life-history parameters, thereby facilitating the research on the ecology, population dynamics, and pest management of *P. striolata*.

## Figures and Tables

**Table 1:** The information on *Phyllotreta striolata* populations.

Population	Location of the Parental Population	Latitude, Longitude, Elevation	The Host Plant of the Parental Population
LF	Langfang, Hebei	39.51° N, 116.61° E, 18.0 m	*Brassica rapa* var. *chinensis*
SH	Fengxian, Shanghai	30.90° N, 121.41° E, 5.0 m	*Brassica rapa* var. *chinensis*
NJP	Nanjing, Jiangsu	32.04° N, 118.87° E, 50.0 m	*Raphanus sativus* var. *longipinnatus*
NJ10	Nanjing, Jiangsu	32.04° N, 118.87° E, 50.0 m	*Brassica rapa* var. *glabra*
NJ11	Nanjing, Jiangsu	32.04° N, 118.87° E, 50.0 m	*Brassica rapa* var. *chinensis*
NJ *	Nanjing, Jiangsu	32.04° N, 118.87° E, 50.0 m	*Raphanus sativus* var. *longipinnatus*

*: NJ is the parental population of NJF1, NJF2, and NJF3.

**Table 2:** The developmental durations and survival rates of *Phyllotreta striolata* across four developmental stages in three laboratory-rearing consecutive generations.

Stage	Population *	Duration (Day)		Survival Rate (%)	
		n1	Mean ± SE	n2	Mean ± SE
Egg	NJF1	202	4.90 ± 0.06 A	5	49.63 ± 5.85 a
	NJF2	111	3.81 ± 0.07 B	4	29.58 ± 5.19 b
	NJF3	114	3.44 ± 0.06 C	3	34.62 ± 5.16 ab
Larva	NJF1	96	11.13 ± 0.21 A	5	62.41 ± 6.77 a
	NJF2	28	11.18 ± 0.39 A	4	27.38 ± 7.84 b
	NJF3	42	11.36 ± 0.32 A	3	37.04 ± 7.49 b
Prepupa	NJF1	78	2.47 ± 0.10 B	5	81.93 ± 4.81 a
	NJF2	18	3.39 ± 0.24 A	3	63.64 ± 11.68 ab
	NJF3	22	2.91 ± 0.20 AB	3	52.50 ± 8.99 b
Pupa	NJF1	53	5.36 ± 0.11 A	5	70.59 ± 7.51 a
	NJF2	9	4.56 ± 0.24 B	3	50.00 ± 18.16 a
	NJF3	13	5.31 ± 0.22 AB	3	57.14 ± 14.67 a

For the same stage, mean ± SE (Standard Error) values within a column followed by the same uppercase or lowercase letter (indicating duration or survival rate, respectively) are not significantly different among the generations (*p* > 0.05). *: Initial three consecutive generations of *P. striolata* reared in the laboratory. n1: number of individuals. n2: number of groups.

## Data Availability

The original contributions presented in the study are included in the article/Supplementary Material, further inquiries can be directed to the corresponding authors.

## Supplementary Materials

The following supporting information can be downloaded at: https://www.mdpi.com/article/10.3390/insects16030260/s1, Figure S1: The schematic diagram of the rearing device for *Phyllotreta striolata* corresponding to Figure 1; Figure S2: The original photos of *Phyllotreta striolata* at various developmental stages; Table S1: The original data of developmental durations of *Phyllotreta striolata* across four developmental stages in five F1 populations and three generations; Table S2: The original data of survival rates of *Phyllotreta striolata* across four developmental stages in five F1 populations and three generations; Table S3: The original data of three response parameters in F1 *Phyllotreta striolata* populations.
