# Supplementary figures and images for: Life-History Parameters of Phyllotreta striolata (F.) (Coleoptera: Chrysomelidae) Acquired by a Laboratory-Rearing Method
Source: Insects. 2025 Mar 3;16(3):260. doi: 10.3390/insects16030260 (PMC11942738; doi:10.3390/insects16030260)

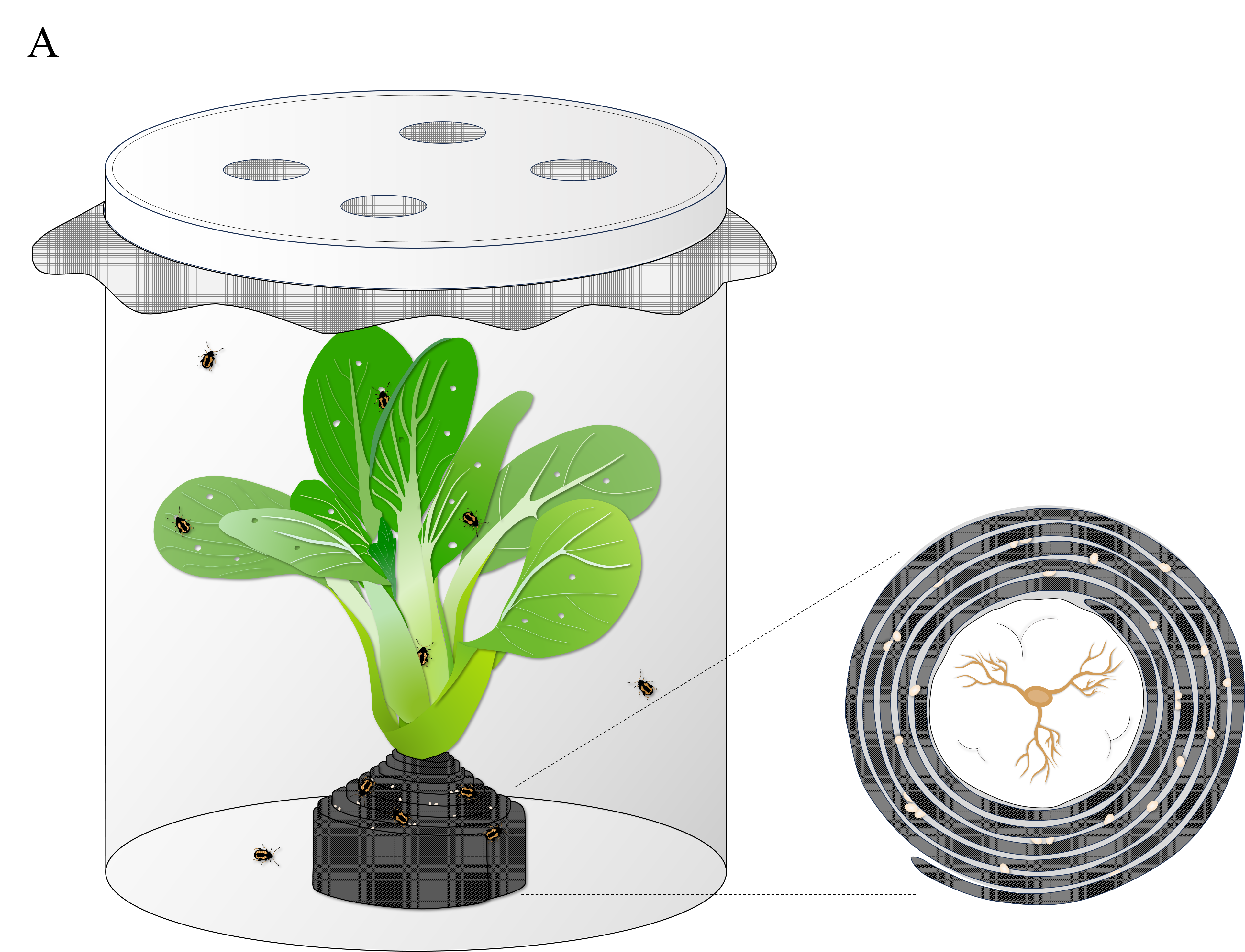

Supplement: Supplementary file 1 [file insects-16-00260-s001.zip › Figure S1-The schematic diagram of the rearing device for Phyllotreta striolata/Figure S1A-The device for adult rearing and oviposition.tif]

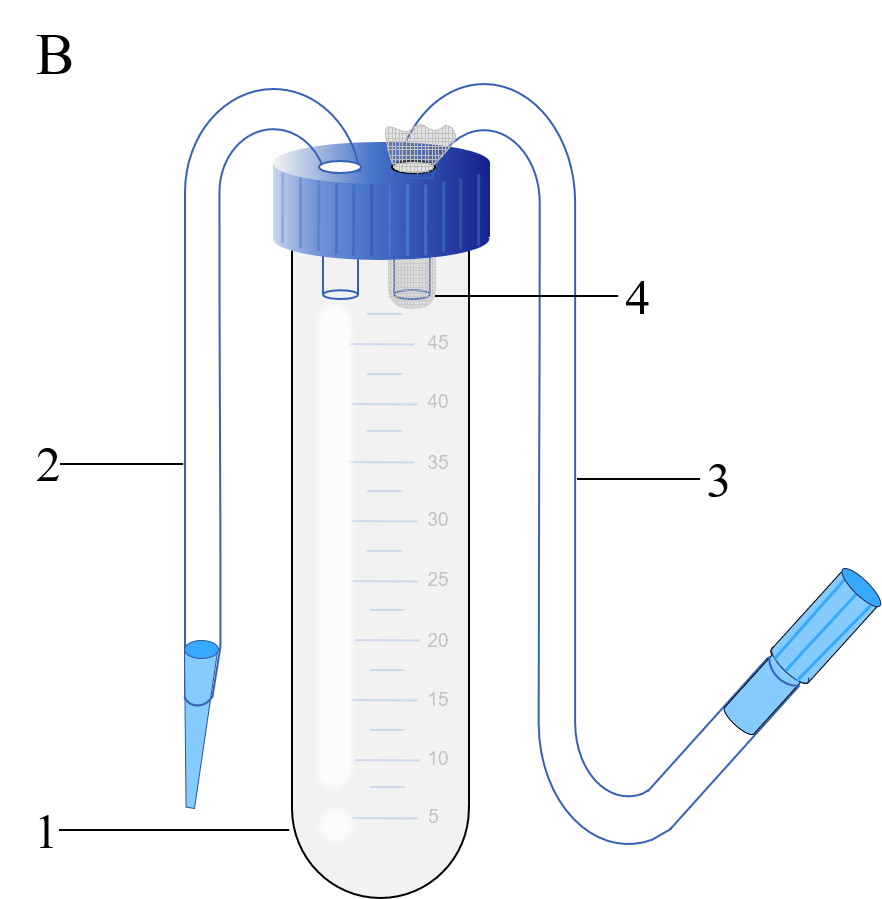

Supplement: Supplementary file 1 [file insects-16-00260-s001.zip › Figure S1-The schematic diagram of the rearing device for Phyllotreta striolata/Figure S1B-The suction trap device for adult collection.tif]

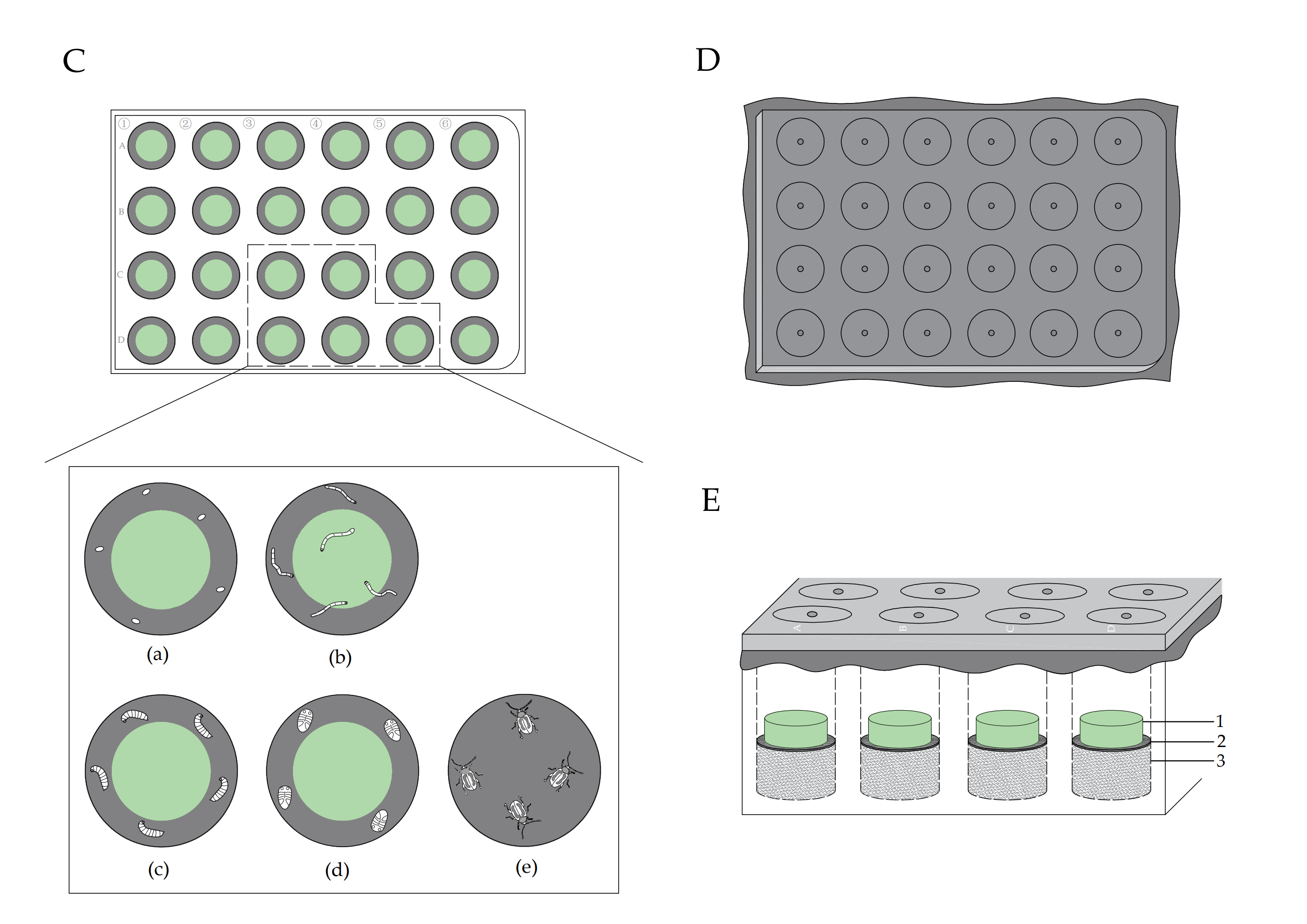

Supplement: Supplementary file 1 [file insects-16-00260-s001.zip › Figure S1-The schematic diagram of the rearing device for Phyllotreta striolata/Figure S1C-1E-The device for rearing immature stages.tif]

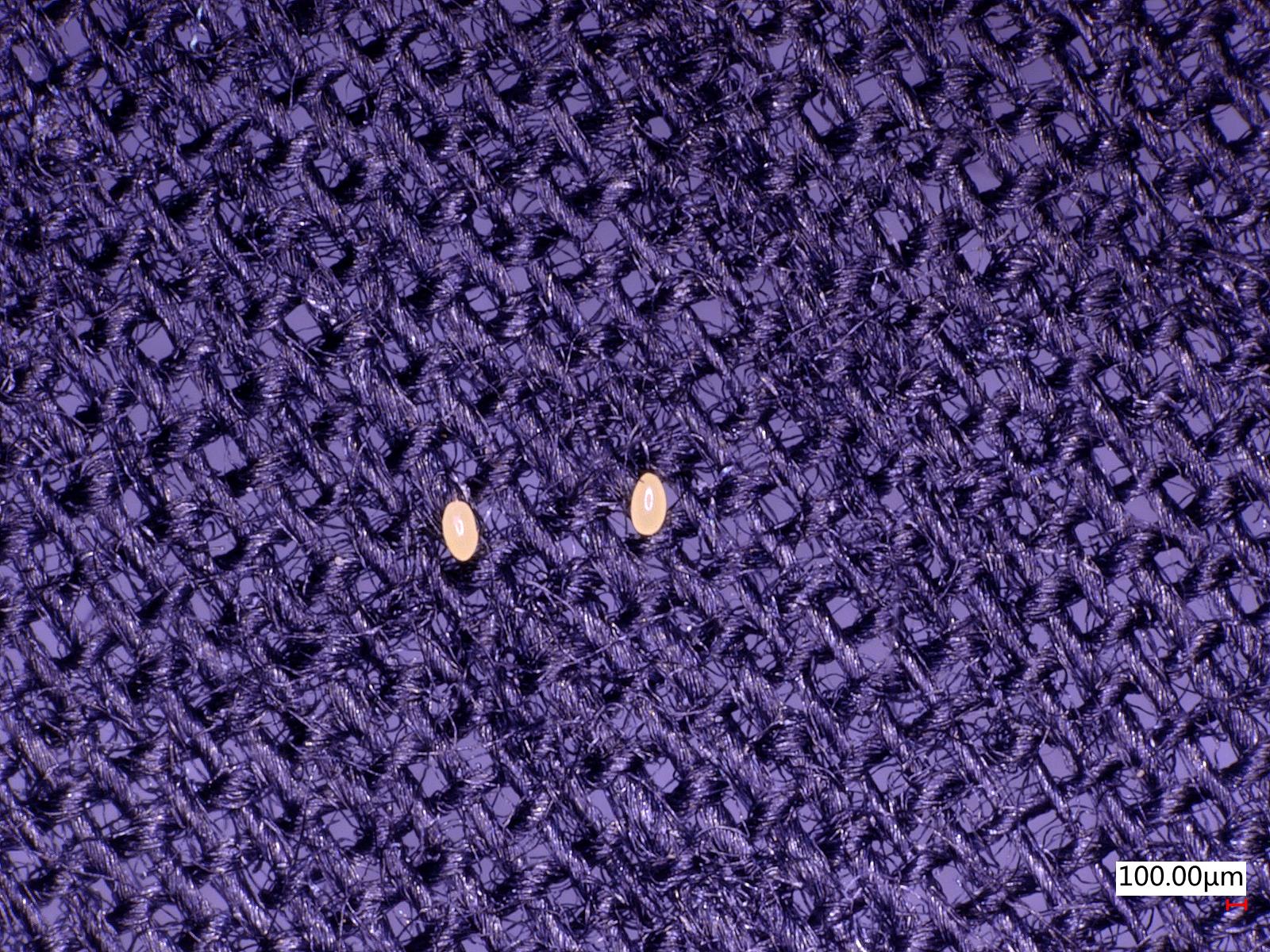

Supplement: Supplementary file 1 [file insects-16-00260-s001.zip › Figure S2-The original photos of Phyllotreta striolata at various developmental stages/A-Eggs.jpg]

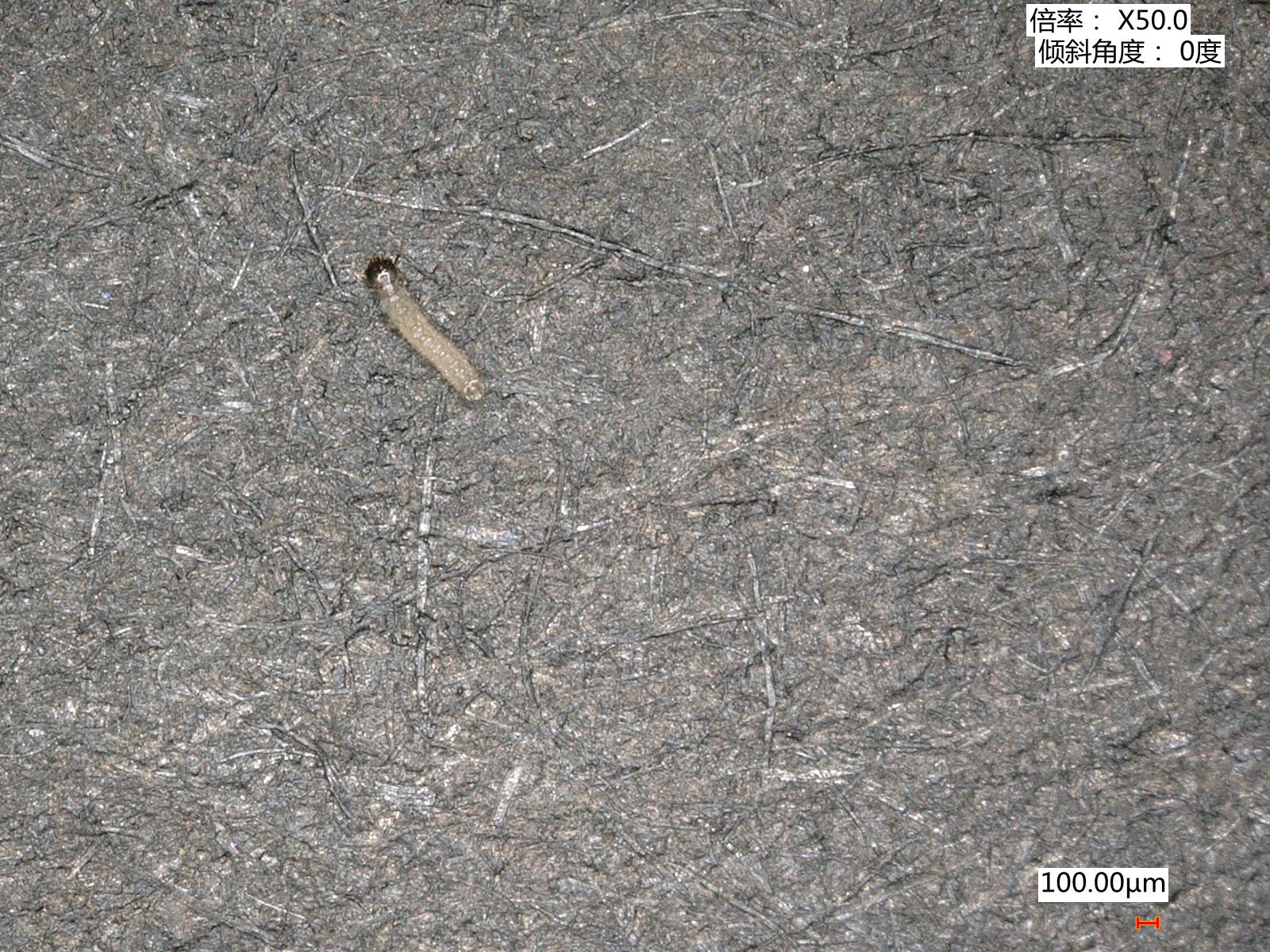

Supplement: Supplementary file 1 [file insects-16-00260-s001.zip › Figure S2-The original photos of Phyllotreta striolata at various developmental stages/B-1st instar larva.jpg]

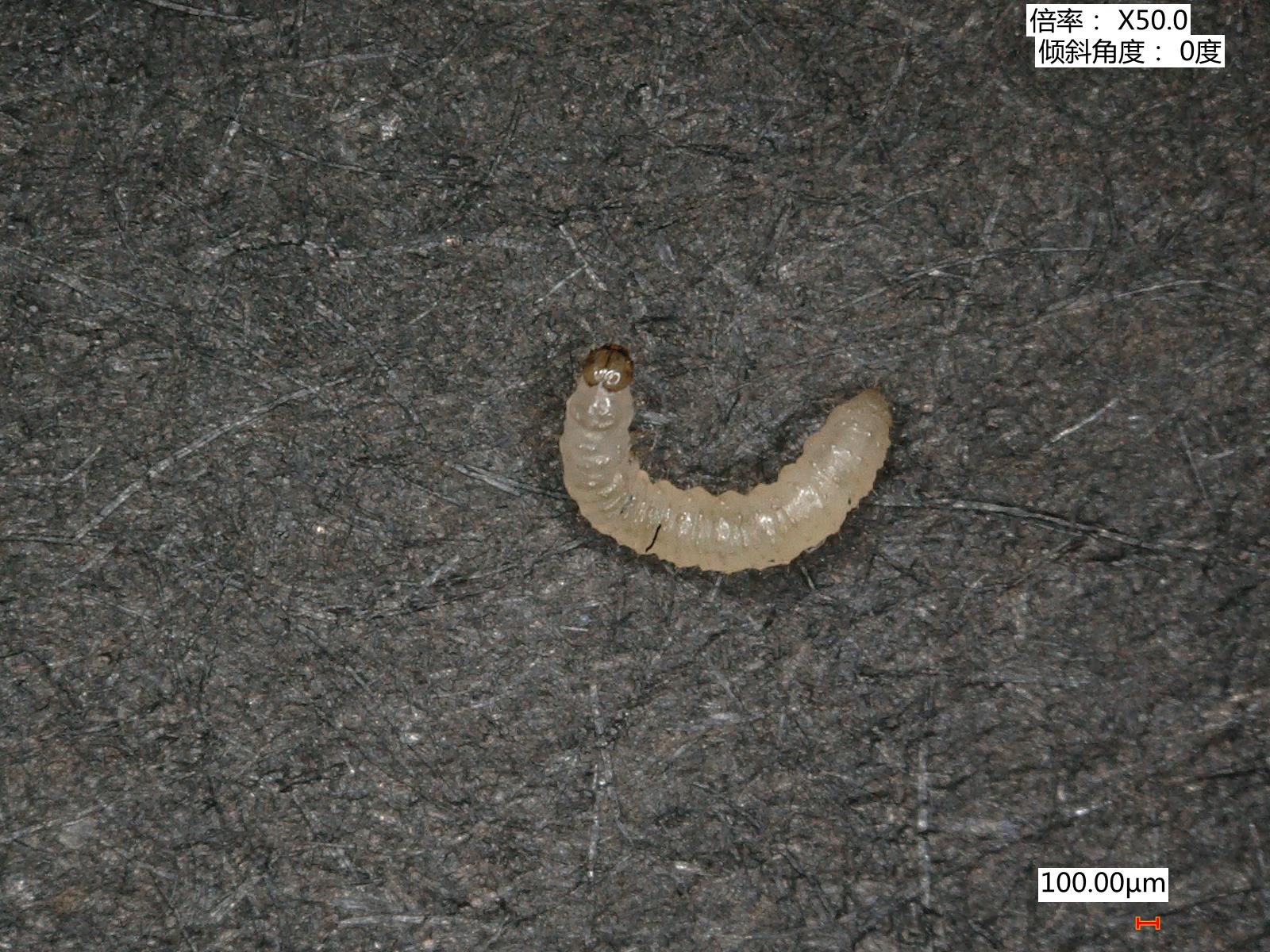

Supplement: Supplementary file 1 [file insects-16-00260-s001.zip › Figure S2-The original photos of Phyllotreta striolata at various developmental stages/C-2nd instar larva.jpg]

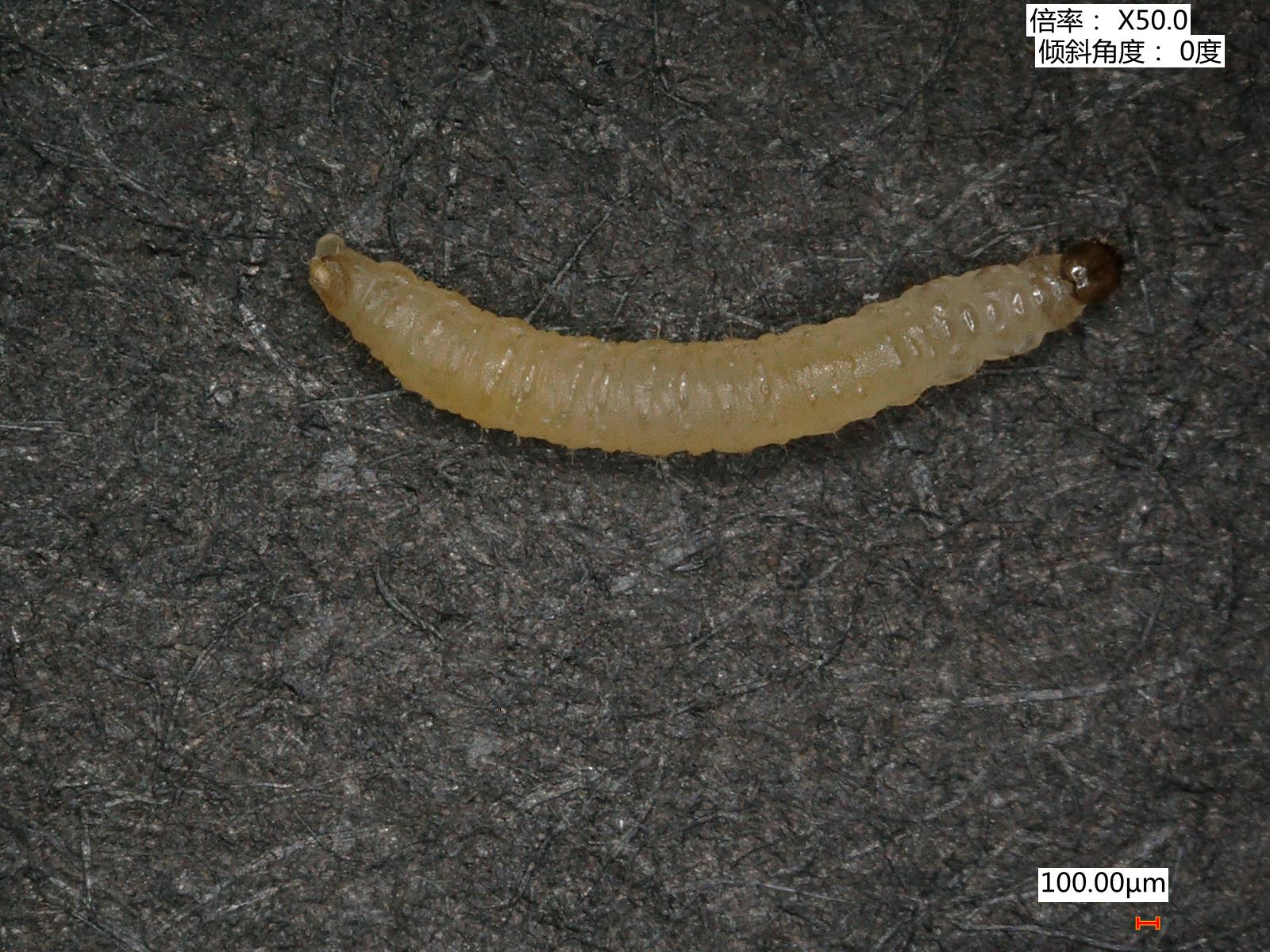

Supplement: Supplementary file 1 [file insects-16-00260-s001.zip › Figure S2-The original photos of Phyllotreta striolata at various developmental stages/D-3rd instar larva.jpg]

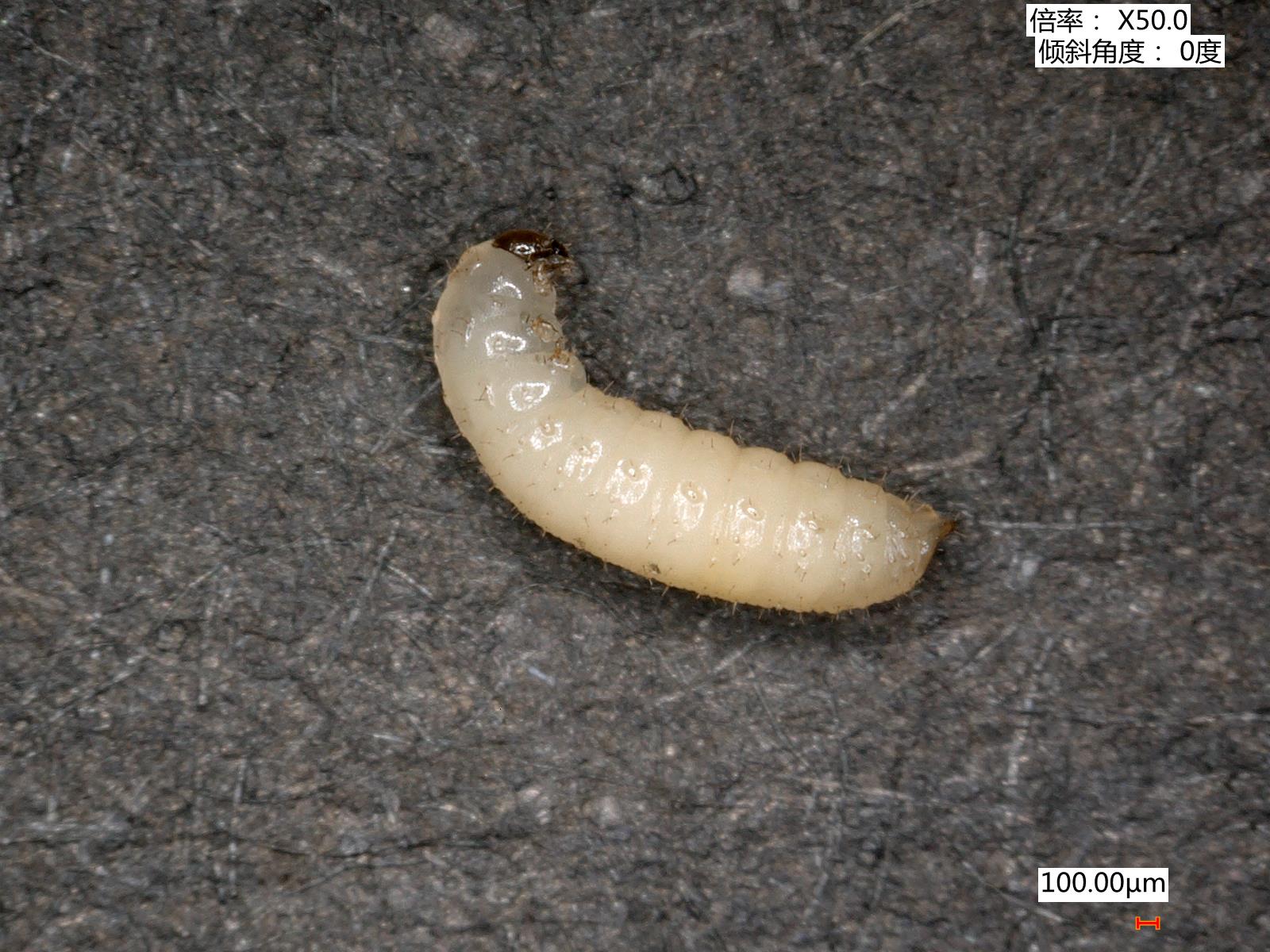

Supplement: Supplementary file 1 [file insects-16-00260-s001.zip › Figure S2-The original photos of Phyllotreta striolata at various developmental stages/E1-Prepupa (side view).jpg]

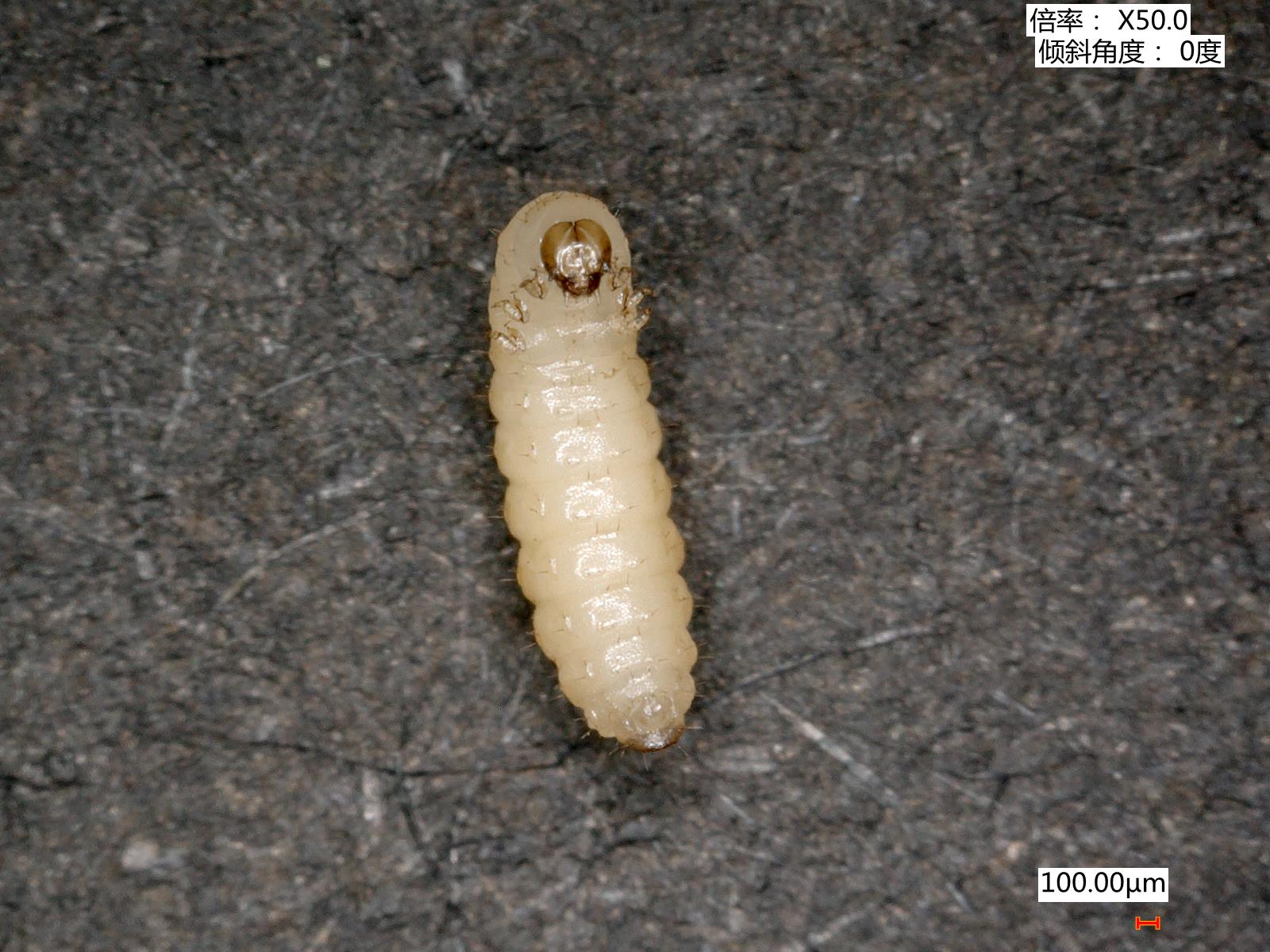

Supplement: Supplementary file 1 [file insects-16-00260-s001.zip › Figure S2-The original photos of Phyllotreta striolata at various developmental stages/E2-Prepupa (ventral view).jpg]

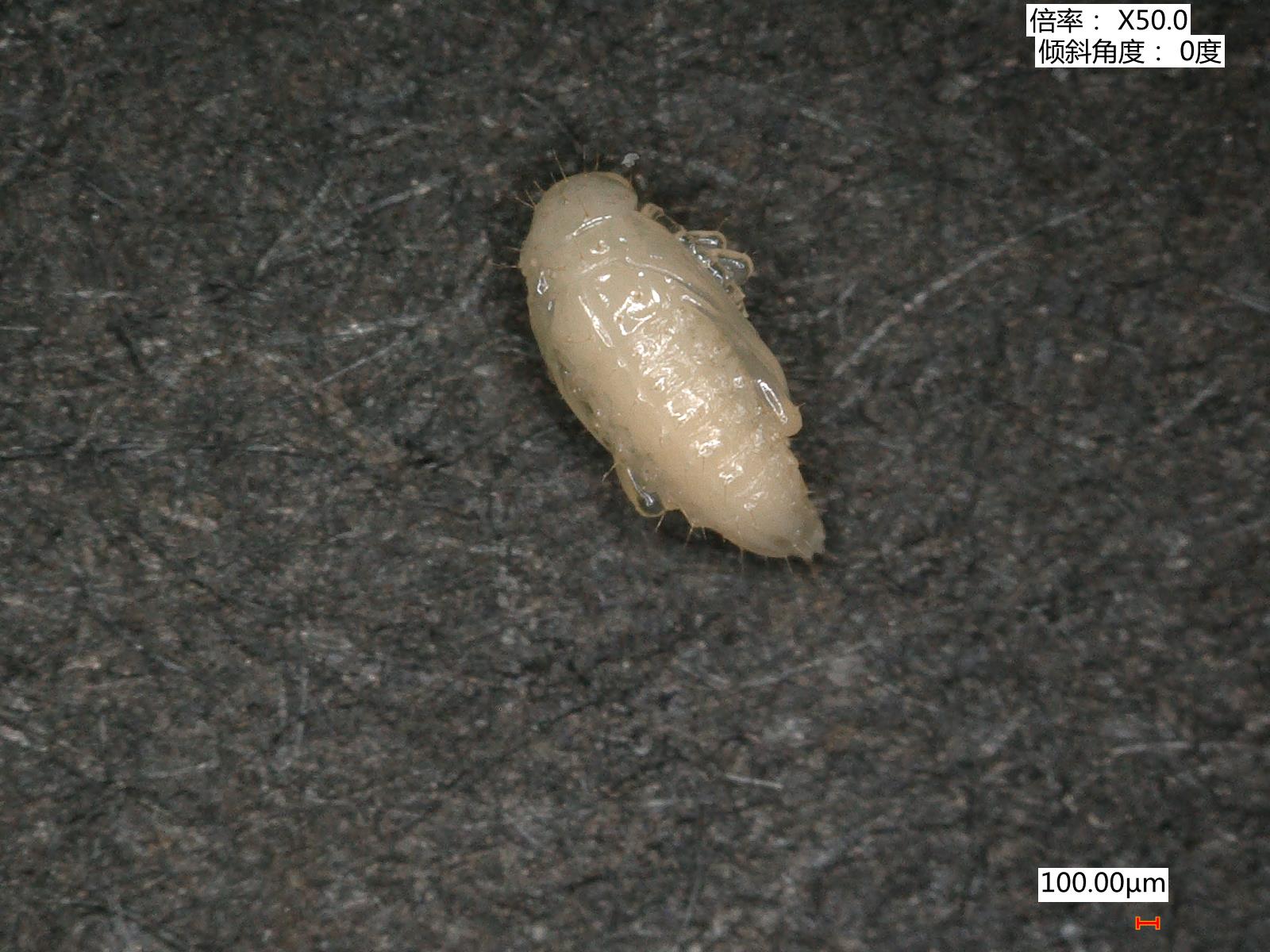

Supplement: Supplementary file 1 [file insects-16-00260-s001.zip › Figure S2-The original photos of Phyllotreta striolata at various developmental stages/F1-Pupa (dorsal view).jpg]

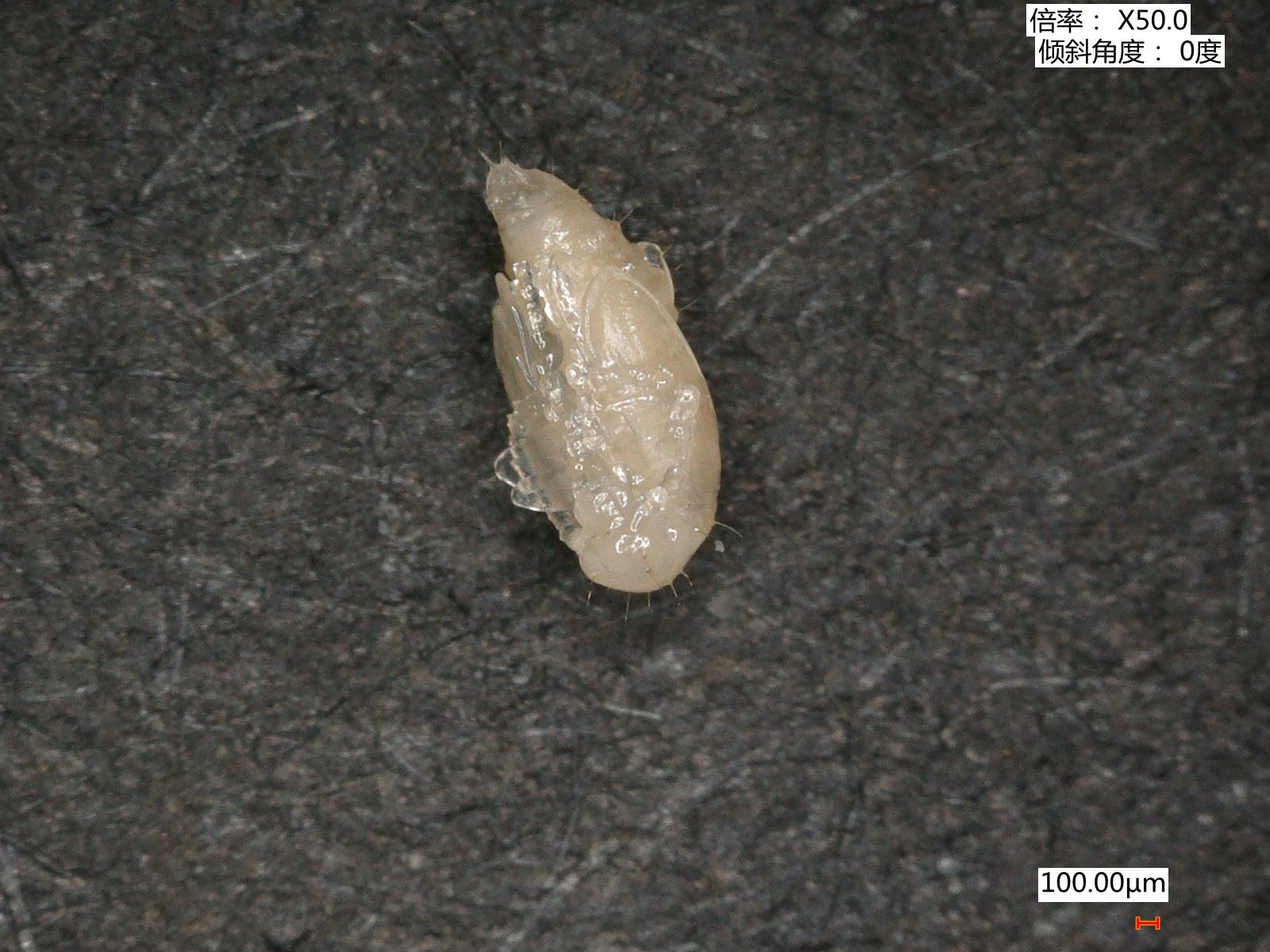

Supplement: Supplementary file 1 [file insects-16-00260-s001.zip › Figure S2-The original photos of Phyllotreta striolata at various developmental stages/F2-Pupa (ventral view).jpg]

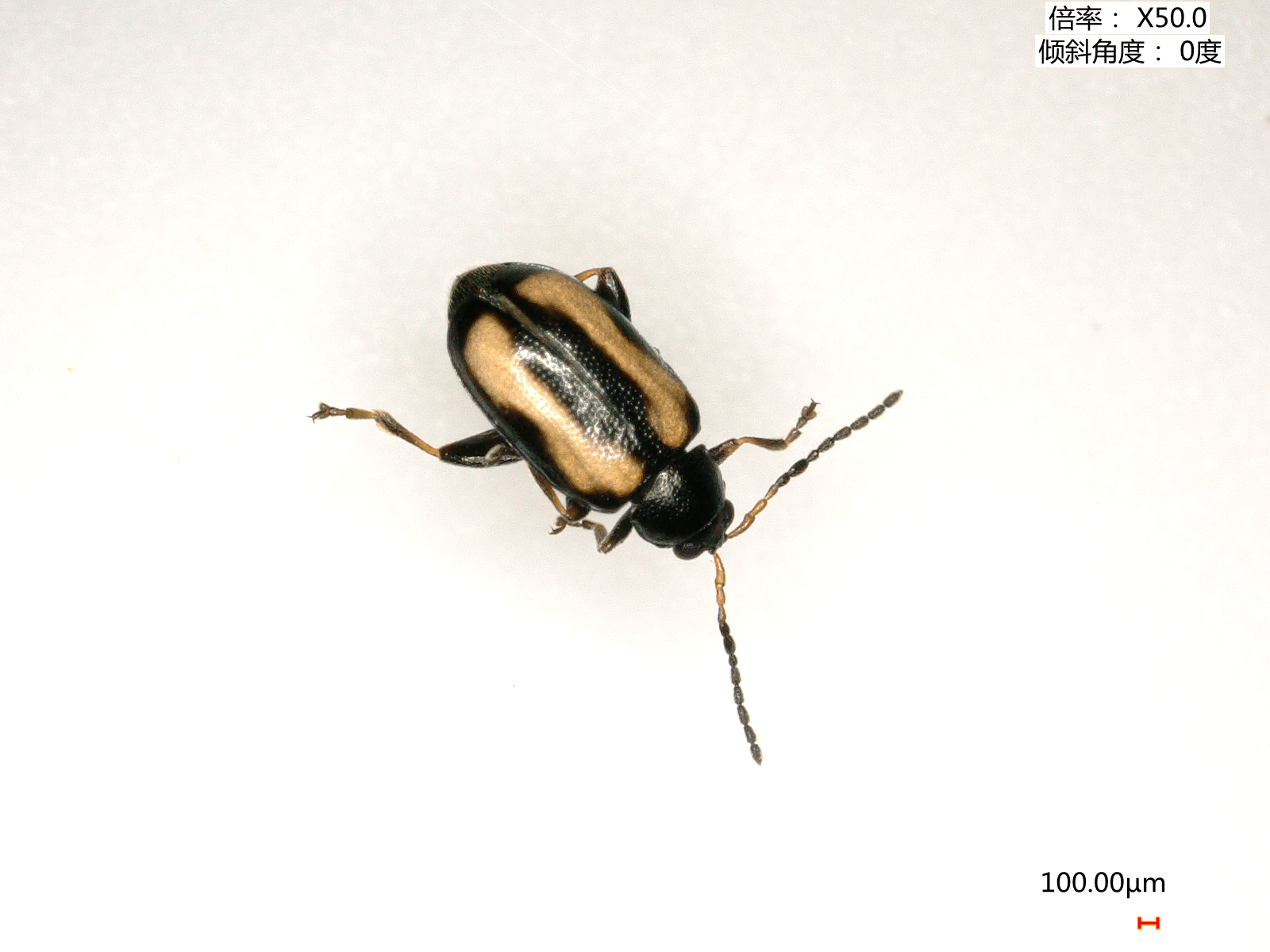

Supplement: Supplementary file 1 [file insects-16-00260-s001.zip › Figure S2-The original photos of Phyllotreta striolata at various developmental stages/G1-Male adult (dorsal view).tif]

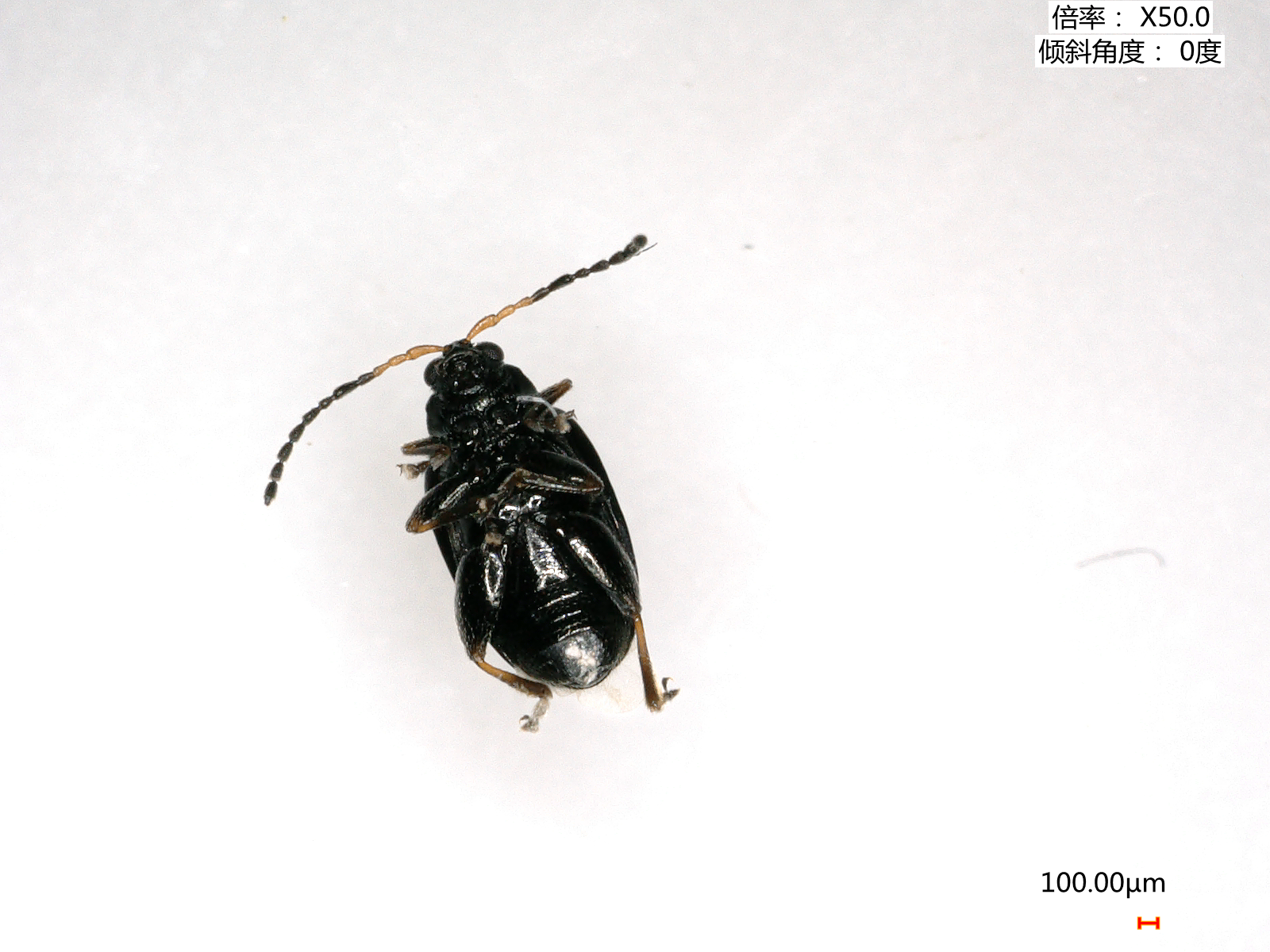

Supplement: Supplementary file 1 [file insects-16-00260-s001.zip › Figure S2-The original photos of Phyllotreta striolata at various developmental stages/G2-Male adult (ventral view).tif]

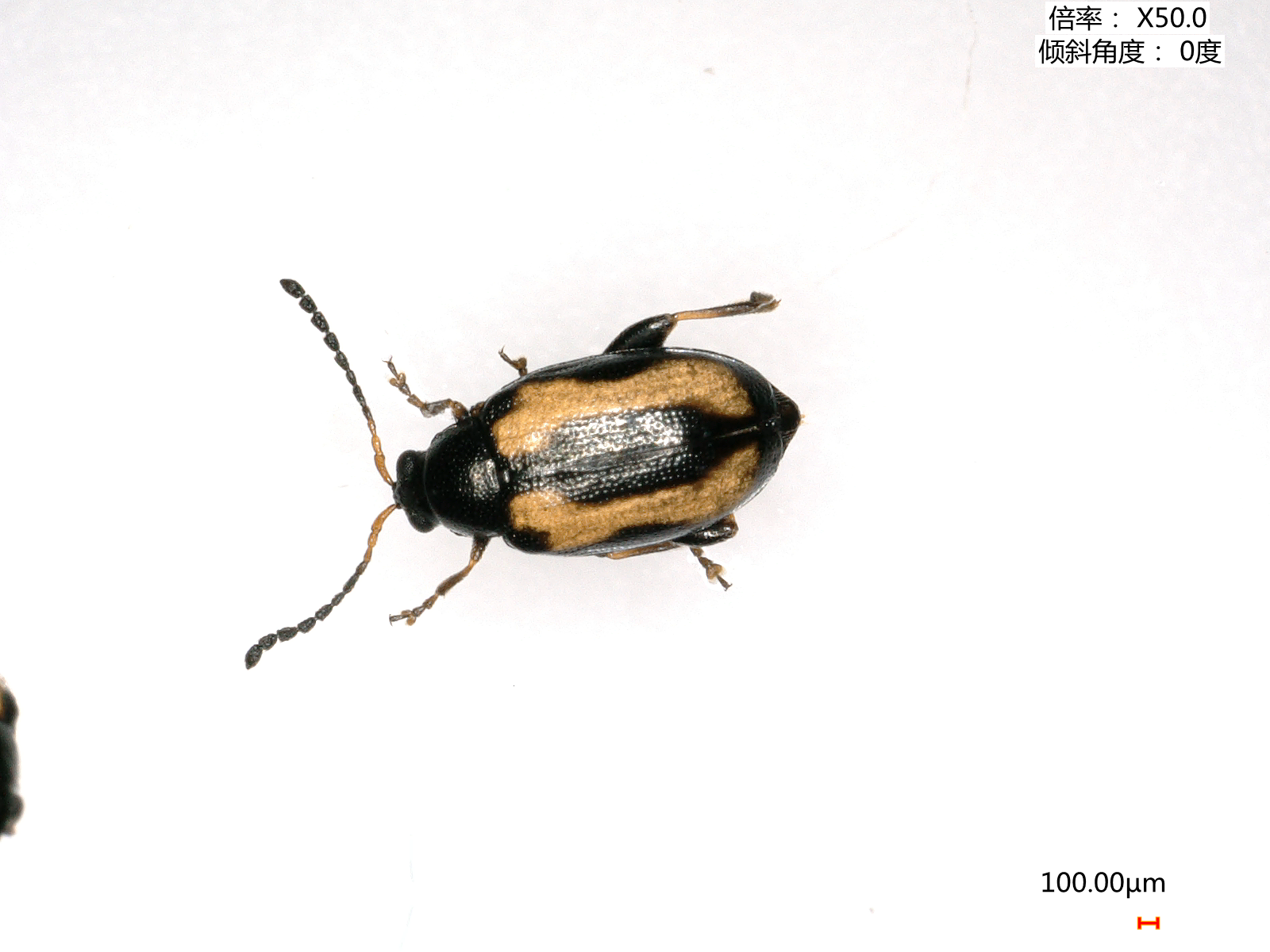

Supplement: Supplementary file 1 [file insects-16-00260-s001.zip › Figure S2-The original photos of Phyllotreta striolata at various developmental stages/H1-Female adult (dorsal view).tif]

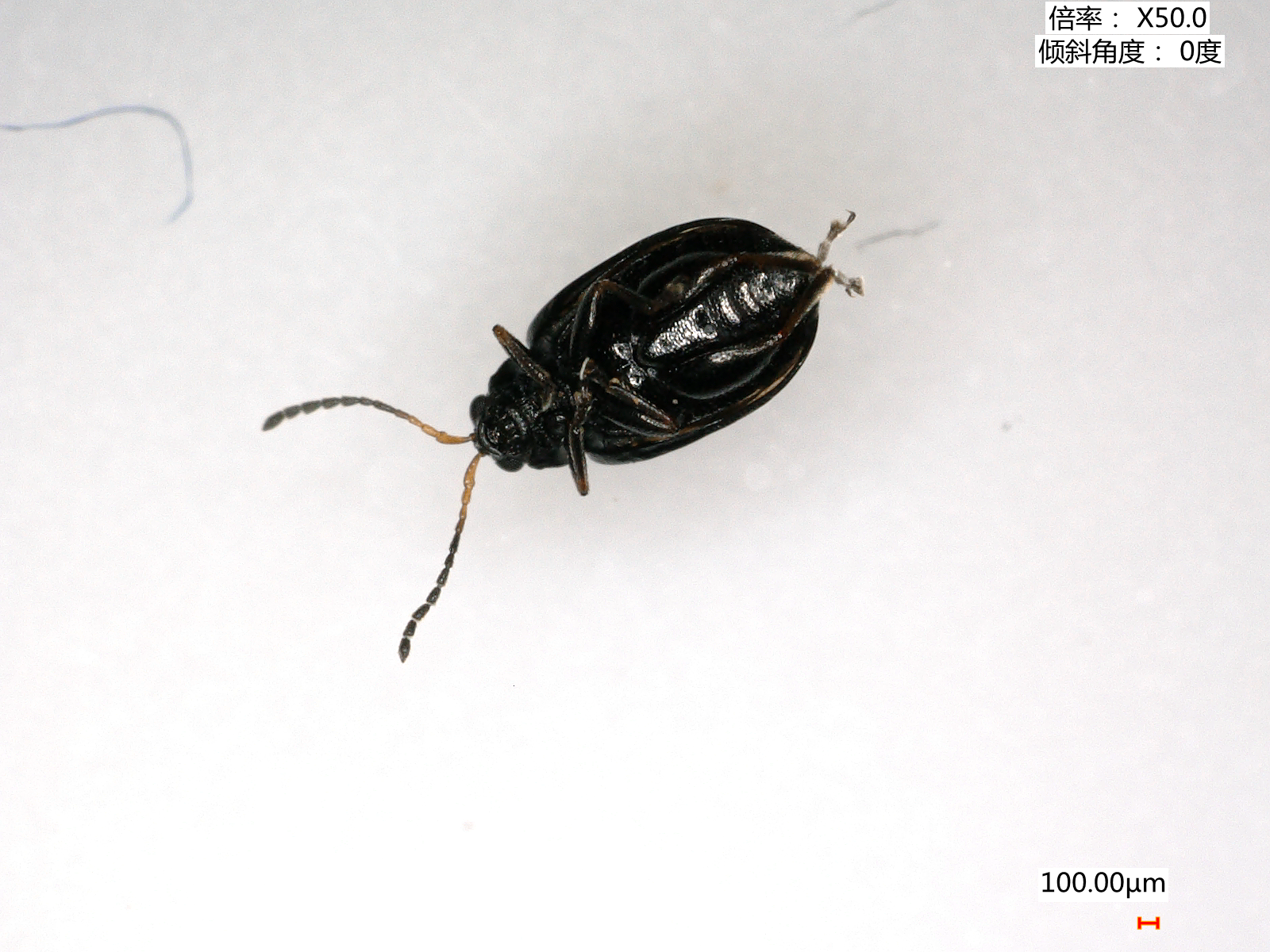

Supplement: Supplementary file 1 [file insects-16-00260-s001.zip › Figure S2-The original photos of Phyllotreta striolata at various developmental stages/H2-Female adult (ventral view).tif]
